# Estimating Power Plant Contributions to Surface Pollution in a Wintertime Arctic Environment

**DOI:** 10.1021/acsestair.5c00030

**Published:** 2025-04-21

**Authors:** Natalie Brett, Steve R. Arnold, Kathy S. Law, Jean-Christophe Raut, Tatsuo Onishi, Brice Barret, Elsa Dieudonné, Meeta Cesler-Maloney, William Simpson, Slimane Bekki, Joel Savarino, Sarah Albertin, Robert Gilliam, Kathleen Fahey, George Pouliot, Deanna Huff, Barbara D’Anna

**Affiliations:** † Sorbonne Université, UVSQ, CNRS, LATMOS, 75252 Paris, France; ‡ Institute for Climate and Atmospheric Science, School of Earth & Environment, University of Leeds, Leeds LS2 9JT, United Kingdom; § Laboratoire d’Aérologie (LAERO), Université Toulouse III − Paul Sabatier, CNRS, 31400 Toulouse, France; ∥ Laboratoire de Physico-Chimie de l’Atmosphère (LPCA), Université du Littoral Côte d’Opale (ULCO), 59140 Dunkirk, France; ⊥ Geophysical Institute and Department of Chemistry and Biochemistry, University of Alaska Fairbanks, Fairbanks, Alaska 99775, United States; # Univ. Grenoble Alpes, CNRS, IRD, INRAE, Grenoble INP, IGE, 38000 Grenoble, France; ∇ Center for Environmental Measurement and Modeling, Office of Research and Development, US EPA, Research Triangle Park, North Carolina 27709, United States; ○ Alaska Department of Environmental Conservation, P.O. Box 111800, Juneau, Alaska 99811-1800, United States; ◆ Aix Marseille Univ, CNRS, LCE, 13331 Marseille, France

**Keywords:** Local Arctic emissions, power plants, stable boundary layer, ALPACA-2022, winter pollution

## Abstract

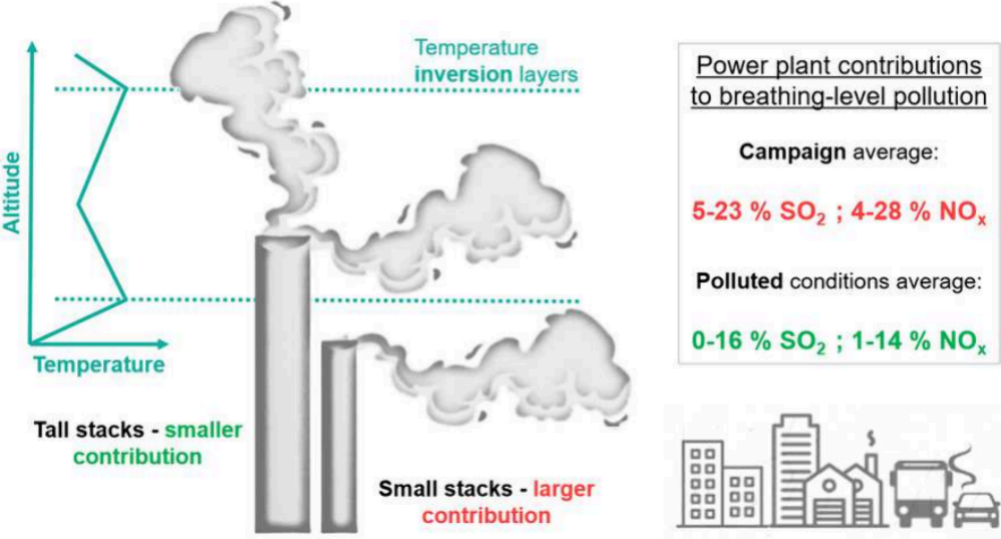

Arctic winter meteorology and orography in the Fairbanks North Star Borough (FNSB, interior Alaska) promote stably stratified boundary layers, often causing acute pollution episodes that exceed the US-EPA National Ambient Air Quality Standards. Power plant emission contributions to breathing level (0–10 m) pollution are estimated over the FNSB using high-resolution Lagrangian tracer simulations run with temporally varying emissions and power plant plume rise accounting for atmospheric boundary layer stability and validated against comprehensive ALPACA-2022 observations. Average relative power plant contributions of 5–23% and 4–28% are diagnosed for SO_2_ and NO_*x*_, respectively, with lower relative contributions in polluted conditions due to larger surface emissions. Highest population-weighted contributions are found in central and eastern (residential) areas of Fairbanks. Significant temporal variability in power plant contributions is revealed, depending on power plant operations and Arctic boundary layer stability. Vertical transport of power plant tracers to the surface depends on the interplay between the presence of temperature inversion layers and power plant stack heights as well as prevailing large-scale or local winds. Notably, power plant emissions can be transported to the surface even under strongly stable conditions, especially from shorter stacks, whereas down mixing from tall stacks mainly occurs under weakly stable conditions.

## Abstract 2

Model-based estimates of power plant contributions to wintertime Arctic air pollution at breathing level (0–10 m) depend on Arctic boundary layer stability, power plant stack height and temporal variability in the power plant emissions.

## 1 Introduction

Human health and ecosystems in Arctic and sub-Arctic environments are adversely affected by local pollution sources.^1−3^ These problems may be exacerbated in future years through increases in Arctic urbanisation and industrial activities as a result of climate warming and economic growth.^4^ Local Arctic emissions include industrial mining, domestic heating and transportation near the surface, and electricity generation (often cogeneration of heat) from fossil fuel power plants with elevated stacks above the surface level.^5,6^ Studies investigating the influence of emissions from the power generation sector to air pollution in the Arctic are limited, and generally focus on long-range transport of remote sources over different seasons, or struggle to distinguish between remote and local emission sources.^7−10^ In Longyearbyen, Svalbard, a study by Drotikova et al.^11^ found that power plants can be important sources of atmospheric contaminants, and electron microscopy techniques identified coal and diesel markers from power plant emissions during nonsummer months.^10^ However, very few studies have tried to quantify the contribution of local power generation to pollution levels at the surface during winter in Arctic urban environments.^8,12^ At this time of year, substantial emission demands due to extremely cold conditions, and in some cases poor energy distribution networks,^6,13^ together with limited pollution dispersion induced by strongly stable atmospheric boundary layer (ABL) conditions,^14^ contribute to severe surface air pollution episodes. This contrasts to other regions where there have been many studies investigating the impact of power plant pollution, relative to other surface emission sources, on local air quality using both models and observations.^15−25^ Nevertheless, there is a significant spread in diagnosed contributions to surface pollution, from negligible up to around 60% relative to total emission sources at the surface.^16−20,22,23,25^ Many factors contribute to these differences, including variable meteorological conditions, local topography, power plant stack characteristics, and emission controls.

In this study, we focus on quantifying the contribution of elevated power plant emissions to surface pollution in Fairbanks, the Interior of Alaska (United States, U.S.) during wintertime. Fairbanks, situated in the Fairbanks North Star Borough (FNSB), is an example of a sub-Arctic city subject to acute wintertime air pollution episodes.^26−28^ Hills surrounding the Fairbanks area, to the north, east, and west create a semiopen basin. This topography, in combination with anticyclonic conditions, and extremely cold surface temperatures (as low as −40 °C) induced by strong surface radiative cooling, and limited sunlight, trigger frequent stratification in the wintertime ABL.^29,30^ This stratification leads to formation of surface-based temperature inversions (SBIs) and elevated temperature inversion (EI) layers, which hinder horizontal and vertical dispersion of pollutants.^26,31^ In 2017, part of the FNSB was designated a ’serious nonattainment area’ by the U.S. Environmental Protection Agency (EPA), for regularly exceeding national air quality standards (35 μg m^–3^ PM_2.5_, 24-h average).^32^ Only very limited studies have explicitly examined power plant emissions as part of their analysis of air quality in Fairbanks. Tran and Mölders^27^ evaluated relationships between meteorological and PM_2.5_ surface measurements, concluding that power plant emissions emitted into elevated layers cannot be a major contributor to ground-level pollution because days with the highest average PM_2.5_ surface concentrations occur under calm stagnant conditions. Mölders et al.^31^ included power plant emissions and plume buoyancy calculations in regional WRF-Chem model simulations in Fairbanks and showed that modeled PM_2.5_ was underestimated compared to observations at a single polluted site. However, neither of these studies explicitly investigated power plant contributions to surface pollution and, in particular, at breathing level where poor air quality can affect human health, defined here as the altitude range from 0 to 10 m. A CALPUFF dispersion modeling study, by the Alaskan Department of Environmental Conservation (ADEC), estimated that between 10 and 21% of observed SO_2_, during two short wintertime pollution events in central Fairbanks, could be explained by power plant sources.^12^ In all these studies, the observations used to evaluate the model simulations were extremely limited, essentially making use of data from a single surface monitoring site in central Fairbanks. The very sparse literature exploring the relative influence of fossil fuel power plant emissions to breathing level pollution in Arctic winter raises an important question in terms of local Arctic air quality: **Are emissions emitted by elevated sources contributing appreciably to breathing level air pollution in stably stratified environments during winter?**

To answer this question, we make use of comprehensive observations collected during the Alaskan Layered Pollution and Chemical Analysis field campaign in Fairbanks between January and February 2022 (ALPACA-2022). ALPACA-2022 aimed to investigate processes influencing pollutant emissions, including surface and elevated sources, chemical formation of secondary pollutants in cold, low-photochemistry conditions, and the influence of meteorology on the dispersion of pollutants, including power plants.^33^ Observations were collected at several surface sites together with vertical profile observations at a site in the west of the city.

Model results from a companion paper, Brett et al.,^34^ are used as a basis for the analysis presented here. In that study, model simulations of SO_2_, nitrogen oxides (NO_*x*_) (nitric oxide, NO + nitrogen dioxide, NO_2_) and carbon monoxide (CO) trace gases from surface and elevated power plant sources were evaluated in detail against surface and profile observations.^34^ The results showed that it is important to take into account the presence of ABL SBIs and EIs in the calculation of power plant plume injection since this affects the height of emission relative to trapping by temperature inversions. The model results were also improved based on comparison with observations and a series of sensitivity runs, such as the inclusion of enhanced surface NO_*x*_ emissions from diesel vehicles at very cold temperatures. Here, we use the SO_2_ and NO_*x*_ results from Brett et al.^34^ Both SO_2_ and NO_2_ are criteria pollutants for which the U.S. EPA has set National Ambient Air Quality Standards (NAAQS). Since PM_2.5_ is also a criteria pollutant and the main cause of air quality exceedences in the FNSB during winter, we also use our results to estimate the contribution of primary PM_2.5_ from power plants to breathing level PM_2.5_ in the Fairbanks area.

To our knowledge, this is the first study to investigate the contribution of power plant emissions relative to total (near-surface + power plant) emissions at the breathing level under different meteorological conditions in the Arctic stratified wintertime boundary layer for multiple pollutants and using hourly real-time surface and power plant emission data. The results presented here are also relevant for other wintertime Arctic environments influenced by local anthropogenic emissions, including elevated power plant sources. The methodology is described in Section 2, followed by results and discussion in Section 3. Section 3 discusses the spatial variability in breathing level pollution contributions from power plants and investigates the influence of vertical mixing and stability and power plant stack height on the results. This is followed by examination of average power plant contributions in different areas of Fairbanks. A range of contributions are estimated making use of model sensitivities to power plant plume capping by inversions in the ABL, vertical mixing, and power plant emission magnitudes. The results are also used to estimate primary PM_2.5_ concentrations associated with power plants at the breathing level. In the final section (Section 4), conclusions and perspectives are provided.

## 2 Methodology

This study makes use of FLEXPART-WRF particle dispersion simulations of trace gases for the period of the ALPACA-2022 campaign. The model simulations and emissions are described in detail in the companion study by Brett et al.,^34^ which included validation against ground-based and vertical profile observations, and a series of sensitivity runs to explore causes of model biases. A brief summary is provided here, with more information in Supporting Information (SI) Section S1.1 and Section S1.2. FLEXPART-WRF is driven by WRF simulations generated by the US EPA (EPA-WRF from now on). The emissions used in the model simulations are provided by ADEC for both near surface and elevated emissions by sector, and point source emissions for power plants, at hourly time resolution. They include near-surface emissions such as residential and commercial heating, and transportation sectors, at 1.33 km spatial resolution. Emissions from 8 different power plant stacks (5 facilities) are also included and released as point sources. They were estimated by ADEC based on stack-specific information from the power plant companies about operations during the ALPACA-2022 period. The power plant emissions include fuel-specific emission factors specific to the different stacks and fuel types (coal, diesel and naphtha fuel), as shown in the SI Table S1.

**Figure 1:**
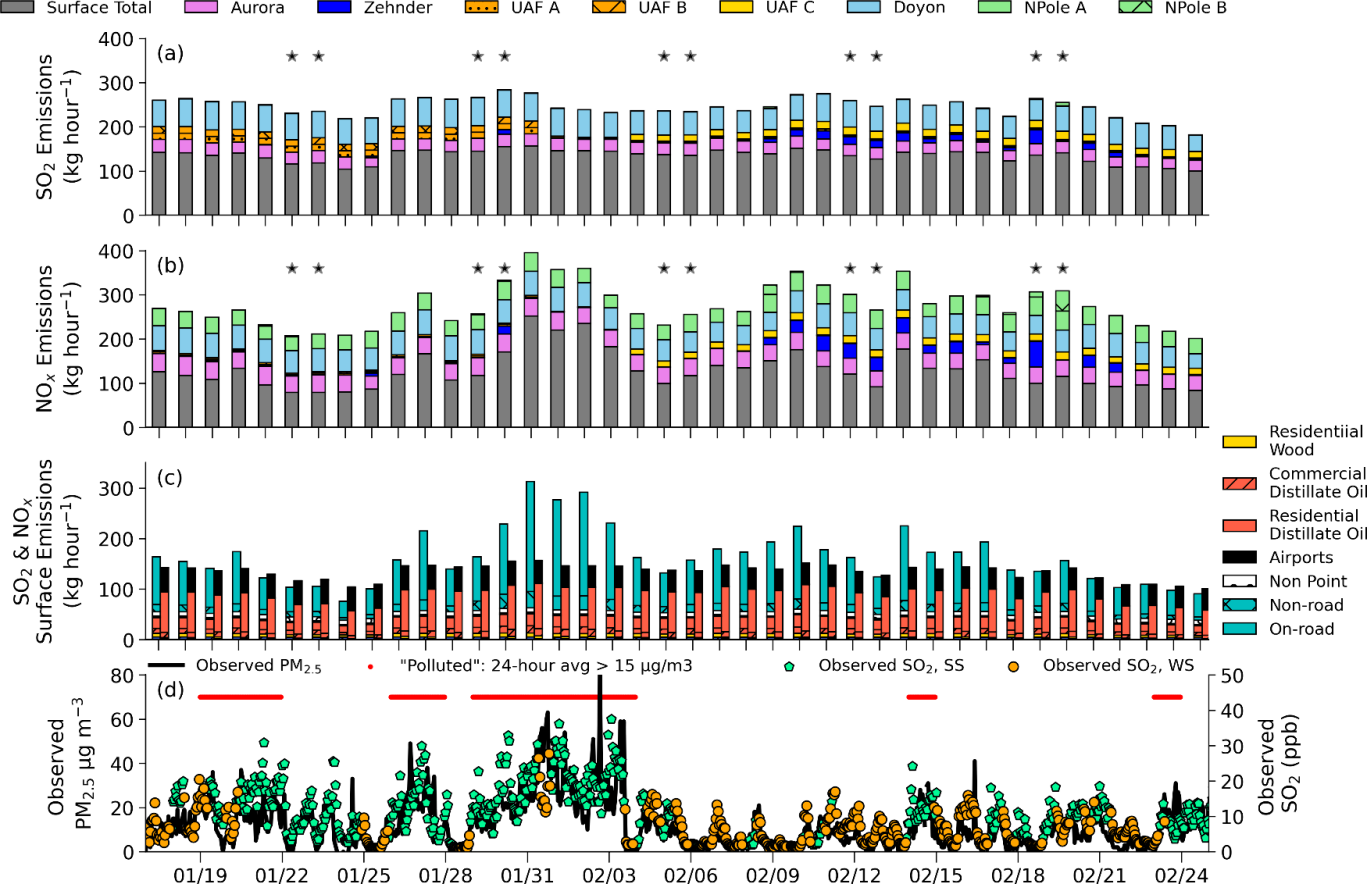
Hourly averaged surface (gray) and power plant emissions (colors) emitted in the FNSB area (kg hour^–1^) each day for (a) SO_2_ and (b) NO_*x*_ according to the ADEC emissions inventory and the emissions from power plants during the ALPACA-2022 campaign. The black stars indicate weekend days on panels a and b. (c) ADEC emission sector contributions (kg hour^–1^) for the total surface emissions for NO_*x*_ (left bars) and SO_2_ (right bars). (d) Left axis: observed hourly PM_2.5_ (μg m^–3^) at the NCore measurement site (Downtown, black line). The ‘polluted’ conditions, used in Section 3, for PM_2.5_ are shown by the thick red lines above. Right axis: observed hourly SO_2_ (parts per billion, ppb) at the CTC measurement site, colored by SS (green) and WS (orange) stability regimes as defined in Brett et al.^34^

Figure 1 (panels a and b) shows the time series of daily near-surface emissions (hourly averaged), for different sectors, from the ADEC emission inventory and individual power plant emissions for each stack, in the FNSB area for SO_2_ and NO_*x*_. Some power plant stacks did not run for the full duration of the campaign, including the three University of Alaska Fairbanks (UAF) stacks and Zehnder, as shown in Figure 1. Differences in SO_2_ and NO_*x*_ power plant emissions occur predominantly due to differences in fuel type and, to some extent, due to emission controls.^34,35^ Detailed evaluation of model simulations against vertical profile observations in Brett et al.^34^ showed that, in addition to the use of temporally varying emissions based on power plant operations, inclusion of a novel plume rise parametrization, including capping of the power plant emissions by near-surface or elevated temperature inversions (if present), led to improved model results. The contributions of different surface emission sectors to SO_2_ and NO_*x*_ emissions during the campaign are shown in Figure 1 (panel c). Surface emission variability is driven by surface temperatures as well as weekday and weekend emission variations. The residential heating sector dominates SO_2_ emissions, while both on-road transport and residential heating are important for NO_*x*_ emissions. Following detailed evaluation against surface observations, model simulations were also improved, as discussed in detail in Brett et al.^34^ Notably, surface NO_*x*_ emissions including an increased cold temperature-dependence for diesel vehicles better reproduces observed surface NO_*x*_ in the model simulations. The results also showed that it is important to take into account conversion of SO_2_ to aerosol phase sulfate, as well as wet and dry deposition of SO_2_ in the model simulations.

The analysis in Brett et al.^34^ showed that meteorology is a key driver of variability in the tracer simulations, although emission variability is important for capturing the observed diurnal cycles at the surface. The model results are also sensitive to the minimum mixing height (*h*_min_) parameter in FLEXPART-WRF since this influences vertical mixing of modeled surface trace gas concentrations (see Section S1.1). Simulation of surface SO_2_ is most affected by *h*_min_ as a large proportion of space heating emissions are emitted above the surface level (5–18 m), while surface CO and NO_*x*_ are predominantly emitted below 5 m. Here, the results using the optimal model setup are used based on the analysis presented in Brett et al.^34^ We also make use of the sensitivity runs with the power plant plume rise capping by SBIs and EIs switched off (NOCAP) and to the vertical mixing (*h*_min_ = 10, 20, and 100 m). Table S2 summarizes the sensitivities and the optimal control (CTRL) setup (capping included and *h*_min_ = 20 m).

**Figure 2:**
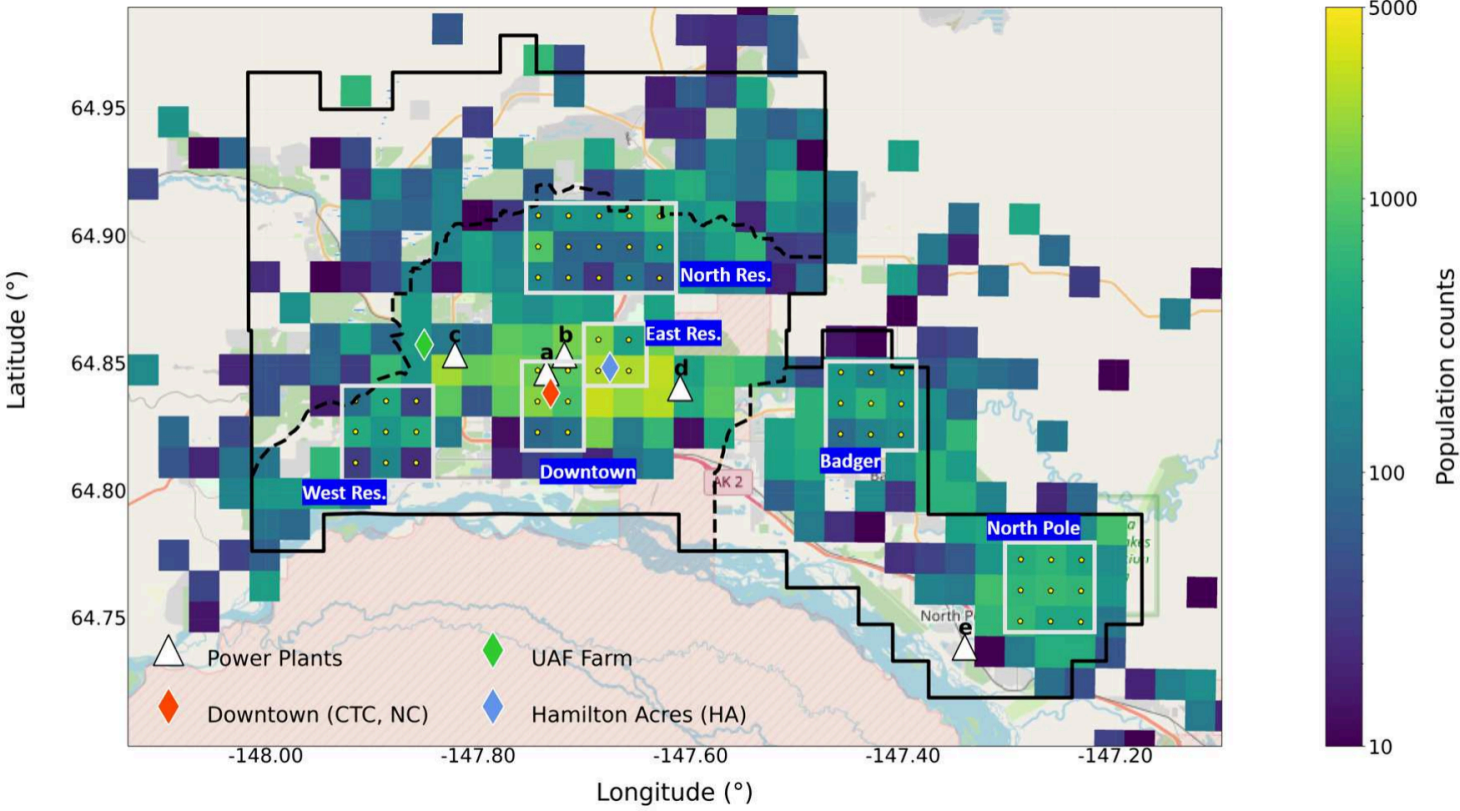
Map of Fairbanks and North Pole. Solid and dashed lines indicate the FNSB and Fairbanks EPA nonattainment areas, respectively.^36^ The power plant locations (white triangles) correspond to the following power plants: (a) Aurora, (b) Zehnder, (c) University Alaska Fairbanks (UAF), (d) Doyon (Fort Wainwright), (e) North Pole. Grid cells (1.33 km resolution) with available population counts are shown. Analysis areas are depicted with the white borders (see text for details). OpenStreetMap contributors 2024. Distributed under the Open Data Commons Open Database License (ODbL) v1.0.

In this study, absolute power plant contributions at breathing level (0–10 m) are estimated from the FLEXPART-WRF tracer simulations as concentration enhancements of power plant tracers above the background. The enhancement of power plant tracer concentrations above background is denoted by δ for SO_2_ and NO_*x*_. The relative contributions with respect to the total tracer (surface plus power plant) at breathing level in the FNSB area are also calculated. Results are examined at several locations in the FNSB (see Figure 2), assigned based on population density and meteorological transport patterns to ensure that a variety of conditions are considered. In line with Brett et al.,^34^ the Downtown area encompasses the UAF Community Technical College (CTC), which was the main ground-based measurement site during ALPACA-2022, and the ADEC monitoring site (NCore), while the East Residential area includes the Hamilton Acres house site, where data on indoor and outdoor pollution was collected during the campaign.^33^ The West Residential Area is located to the south of the UAF Farm and encompasses the Woods residential area. It is located in the southwesterly pollution outflow due to predominant winds from the northeast during anticyclonic conditions.^34^ When conditions are more influenced by large-scale cyclonic weather systems, prevailing southerly winds transport pollution to the north of Fairbanks, including to the North Residential analysis area. Finally, the North Pole area corresponds to the residential area to the southeast of Fairbanks, near the North Pole power plant. Population-weighted contributions (PWCs) for pollutant tracers are also estimated for each area using population counts taken from 4 km gridded resolution census data and mapped onto the 1.33 km grid (see Figure 2).^37^ PWCs are useful for gaining insight into areas that are most affected by power plant pollution in terms of population exposure relative to the FNSB area overall (see Section S1.3 and Table S3 for details).

The power plant contributions to surface pollution levels are also analyzed depending on the meteorological stability near the surface. Stability regimes are defined as in Brett et al.^34^ Hourly PM_2.5_ and SO_2_ observations are shown in Figure 1 (panel d) for both strongly stable (SS) and weakly stable (WS) meteorological regimes, diagnosed based on the strength of surface temperature inversions. SS conditions were more frequent at the beginning of the campaign in January 2022, as shown by the higher observed SO_2_ concentrations. A severe surface pollution episode, due to cold temperatures and SS conditions, occurred from 29 January to 2 February 2022, and is described in more detail in Simpson et al.^33^ and Brett et al.^34^ The results presented in this study are analyzed as averages over the ALPACA-2022 campaign, and for polluted conditions designated using PM_2.5_ concentrations as shown in Figure 1 (panel d). As noted earlier, a range of contributions is estimated using results from selected sensitivity simulations described in Brett et al.^34^ In addition, simulations using ± 50% power plant emissions are also performed. The simulations are summarized in Section S1.2 and Table S2. They provide a range of absolute and relative power plant contributions that take into account uncertainties in dispersion modeling of unique Arctic wintertime conditions.

## 3 Results and Discussion

### 3.1 Spatial Contributions

**Figure 3:**
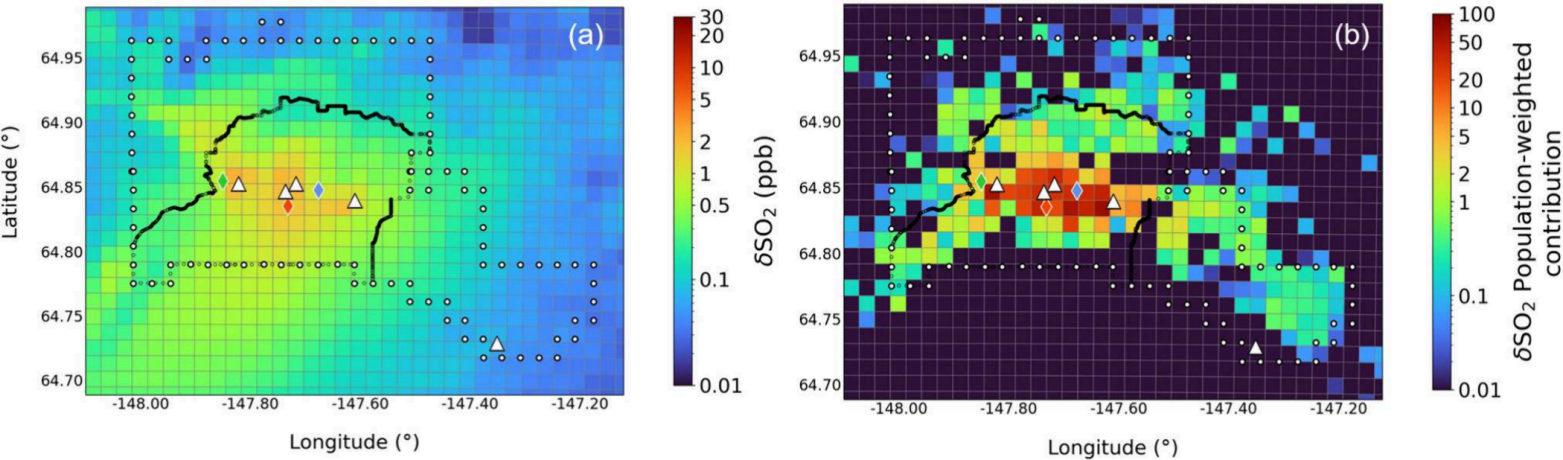
(a) Total δSO_2_ (ppb) power plant enhancements (ppb) at 0–10 m (campaign average). (b) Total SO_2_ power plant population-weighted contributions (PWCs), see Section S1.3 for details. The measurement sites are indicated by the colored diamonds and power plants shown by the white triangles, as in Figure 2.

The total δSO_2_ power plant enhancements between 0 and 10 m, averaged over the full campaign, are shown in Figure 3a. Higher concentrations (up to 5 ppb) are simulated in central Fairbanks, within the nonattainment area borders, with some outflow to the southwest, as also discussed in Brett et al.^34^Figure 3b shows PWCs for each grid cell containing population data (colored boxes in Figure 2). PWCs are much larger over central Fairbanks, predominantly over the Downtown and East Residential areas and to the south of the UAF Farm measurement site (west Fairbanks). In these areas, higher PWC values, due to higher population densities, demonstrate increased population exposure to power plant pollution with respect to the FNSB region. Four of the power plants are located nearby the areas most affected by the power plant emissions, according to the model results. This suggests that the proximity of power plant facilities to the Fairbanks population is suboptimal.

**Figure 4:**
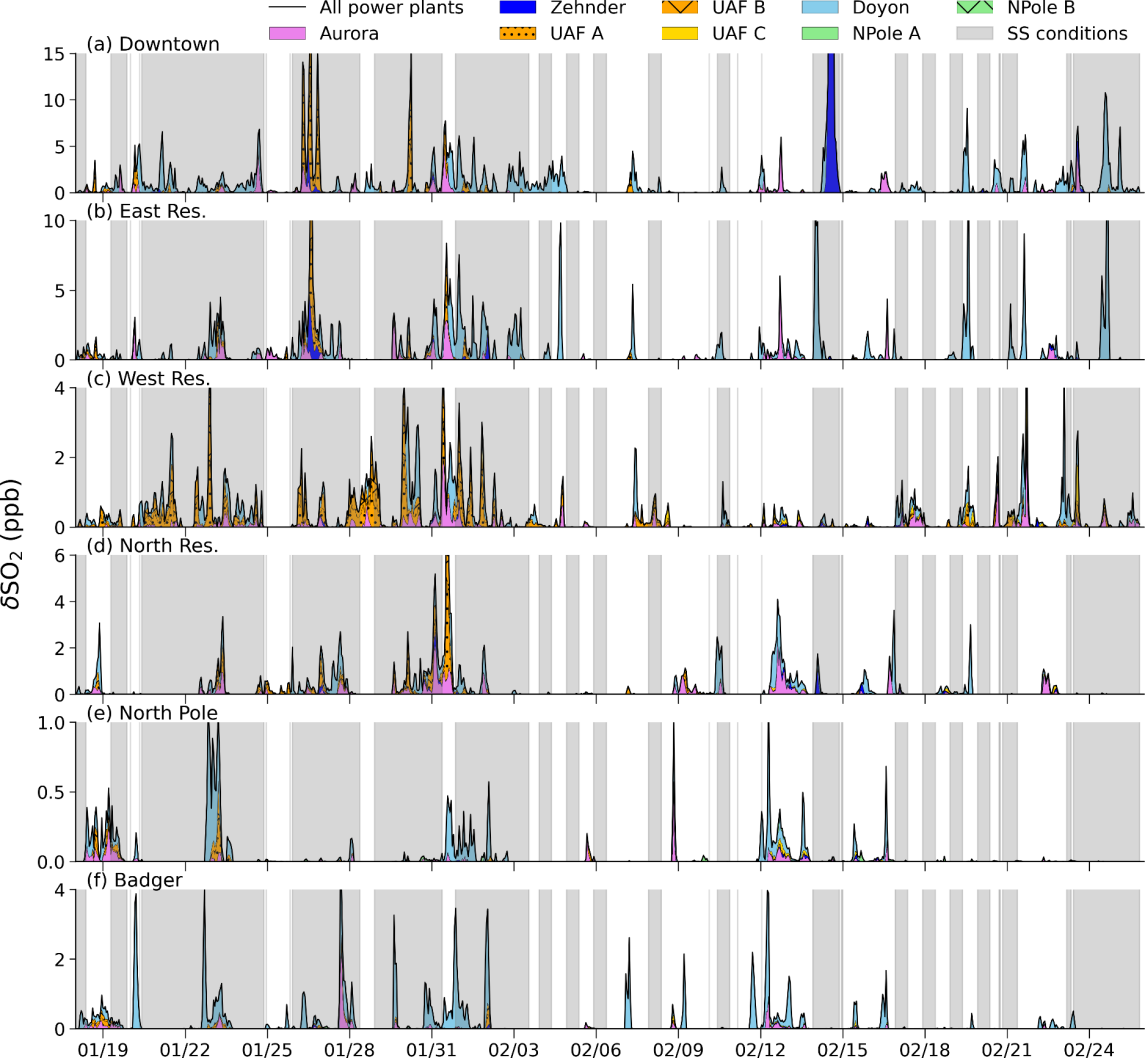
Total power plant δSO_2_ (ppb) enhancements (black lines) between 0 and 10 m as a function of time (Alaskan Standard Time, AKST), colored by the different power plant contributions, indicated in the legend. Panels (a) to (f) correspond to the different analysis areas, indicated in Figure 2. The gray shaded periods correspond to SS conditions, and the nonshaded periods correspond to WS conditions. See text for details.

Figure 4 shows absolute breathing level δSO_2_ contributions from each power plant stack as a function of time, for each area indicated in Figure 2 (δNO_*x*_ provided in SI, Figure S1). SS and WS stability conditions are also indicated. Power plant δSO_2_ contributions are the largest in the Downtown and East Residential areas, primarily from the Doyon and Aurora stacks. Contributions are attributed to individual power plants e.g. Doyon on 2–3 February (Downtown) or to a combination of power plants, e.g. UAF A, Aurora and Zehnder on 27 January (East residential). A strong contribution (>15 ppb δSO_2_) from Zehnder in the Downtown area (Figure 4, panel a) on 14 February is explained by significant Zehnder emissions, stable meteorological conditions on this day (see Figure 1), and the short stack height of Zehnder (18 m). However, such occurrences are infrequent because Zehnder ran intermittently (Figure 1 and Section S1.2).

The main wind directions bringing power plant tracers to each analysis area are also examined. Total power plant contributions are plotted as a function of wind direction and speed, at approximately 40 m altitude (from EPA-WRF simulations) for each area, for δSO_2_ and δNO_*x*_ (see Figure S2 and Section S2 for more details). The North and East Residential areas are influenced by power plant tracers transported from the south/southwest and the east/northeast. In contrast, the Downtown and West Residential contributions mostly originate from the east/northeast (Figure S2). The low UAF A and B stacks (20 m) that ran more often from 18 January to 1 February,^34^ have substantial influences on the West Residential area during this time for δSO_2_ (Figure 4) and δNO_*x*_ (Figure S1). UAF C was the main UAF stack running from 4 February onward when emissions were much greater than UAF A and B. This coincides with reduced δSO_2_ and δNO_*x*_ contributions at 0–10 m due to the taller UAF C stack height (64 m), and possible influences from the local drainage flow at the UAF Farm, limiting vertical mixing.^38^ Lower δSO_2_ and δNO_*x*_ from 4 February onward in this area could also be explained by more stringent emission controls of SO_2_ and NO_*x*_ in the UAF C stack that replaced the UAF A and B stacks.^35^ Although the West Residential area is situated in the dominant southwesterly outflow, this area has lower power plant contributions than Downtown and East Residential areas and smaller PWCs due to lower population density (see Figure 3b).

In the Badger and North Pole areas, winds from the east/northeast lead to small power plant contributions, while winds from the west/northwest and south/southwest result in higher contributions (Figure S2). Both areas are less influenced by power plant SO_2_ (Figure 4) while the North Pole A stack leads to enhanced power plant NO_*x*_ over these areas (Figure S1). However, North Pole A seldom contributes to other areas.

The gray shading in Figure 4 represents SS conditions, and nonshaded areas correspond to WS conditions. The results demonstrate that the simulated power plant tracers are reaching the surface during both SS and WS conditions. The effects of surface stability and stack heights on power plant contributions at breathing level, are explored in more detail in the following sections.

The results presented in this section show that, based on the model simulations, power plant emissions are being transported down to the surface and contribute to breathing-level pollution in the Fairbanks area. The predicted contributions vary widely depending on power plant operations and the meteorological situation. An important finding is that power plant emissions contribute to surface pollution even under very stable meteorological conditions. While ADEC^12^ examined two short pollution episodes, they did not consider power plant influence under variable meteorological conditions, nor the intermittent nature of the contributions from the different power plant stacks.

### 3.2 Cases of Power Plant Emission Transport toward the Surface

In this section, we investigate the influence of boundary layer stability and vertical mixing on power plant contributions at breathing level. This analysis builds on results presented in Brett et al.^34^ which evaluated simulated power plant plumes against vertical profile observations of trace gases collected on-board a Helikite tethered balloon during the campaign for several case studies. As also shown in Figure 4, power plant, tracers are simulated reaching the surface. Here, we evaluate cases of downward transport of power plant tracers to the surface notably under stable meteorological conditions over the Downtown area in central Fairbanks. Model results are compared to the logarithmic form of the range corrected signal (log(RCS)) from wind LiDAR observations (40–290 m),^34,39^ which indicates the presence of aerosol particles, in the absence of snow flakes or ice crystals in clouds,^39^ and provides information about the vertical structure of pollution plumes. We also made use of carbon dioxide (CO_2_) observations at 3 and 23 m at the Downtown CTC site. The difference between CO_2_ at 3–23 m (δCO_2_) provides an indication about vertical mixing toward the surface since CO_2_ is dominated by surface sources, i.e. smaller (larger) delta values indicate more (less) vertical mixing (see discussion in Section S3).

**Figure 5:**
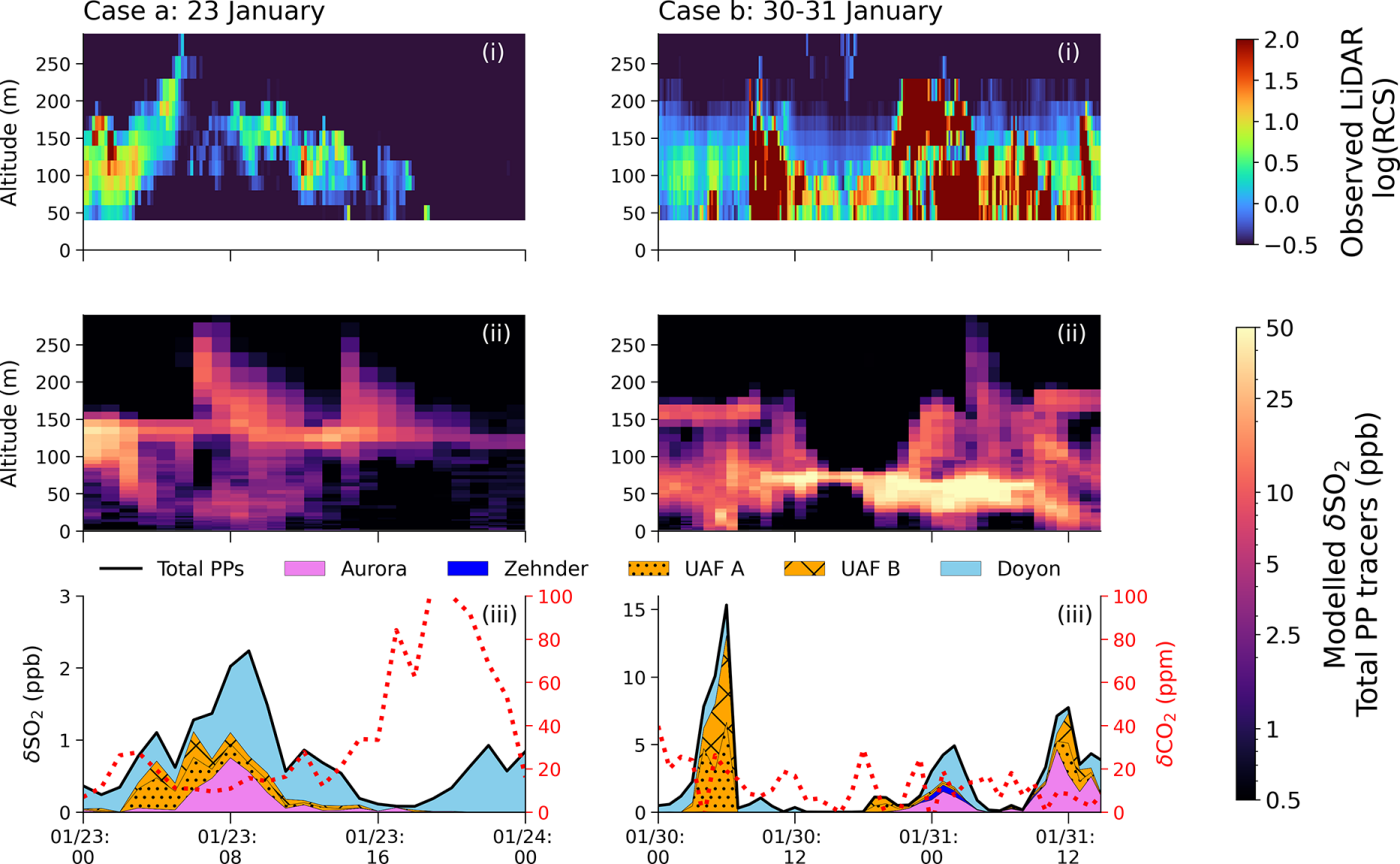
Cases illustrating downward power plant plume transport on (a) 23 January and (b) 30 to 31 January at the CTC site (situated Downtown, see Figure 2), showing (i) log(RCS) of wind LiDAR data as a function of altitude (40–290 m) and time, indicating power plant plumes aloft, (ii) total simulated power plant δSO_2_ (ppb) as a function of altitude (0–290 m) and time (Downtown area), and (iii) total simulated power plant (PP) δSO_2_ (ppb) at 0–10 m (Downtown area), for the stacks indicated (total = black line). The red dotted line corresponds to the 3–23 m δCO_2_ (parts per million, ppm) observed at CTC, see text for details.

Figure 5 shows two cases during SS conditions on January 23 and 30–31 when model results suggest power plant tracers are transported from aloft to breathing level at the Downtown CTC site. This location is subject to large surface emissions from residential/commercial heating and vehicles and intermittent power plant influence (see Figure 4, panel a). In both examples, power plant plume enhancements are simulated with peak concentrations aloft between 50 to 150 m (see panel ii). The simulated plumes have comparable structures to the plumes observed by the wind LiDAR using log(RCS) measurements (panel i). In each case, there was no precipitation, thus the observed backscatter signal most likely corresponds to particles from power plant plumes. There are some differences in simulated and observed plume characteristics that may be explained by the presence of particles that are too small to be observed by the LiDAR (0.5–1.0 μm, 0.7 μm = peak sensitivity),^39^ or because the model output is at lower time resolution (hourly) compared with the wind LiDAR observations (10 min averages). Differences in observed and modeled plume altitudes, such as between 0800 and 1800 Alaskan Standard Time (AKST) on 23 January, may be explained by discrepanices in the plume rise parametrization as discussed in Brett et al.^34^ (see also Section S1.2), or an offset in the timing of the contribution due to differences in modeled versus observed winds. Panel (iii) shows individual power plant δSO_2_ tracer contributions at breathing level as a function of time, together with δCO_2_ for the two examples presented here with SS meteorological conditions.

#### a 23 January

This case is classified as SS, however the surface pollution is lower compared to case (b) (see Figure 1) due to slightly weaker temperature inversions and stronger horizontal wind speeds.^34^ The model simulates power plant plumes that are dispersed downward toward the surface, notably between 0400 AKST and 1400 AKST. Differences in δCO_2_ show a significant variability (5 to >100 ppm) throughout the 24-h period (shown in Figure 5 a, panel (iii). There is an inverse correlation between the δCO_2_ values and the power plant concentrations at 0–10 m. Lower δCO_2_ values suggest mixing within the lowest layers of the ABL, and they are coupled with increased power plant breathing level contributions for this case. Winds from the LiDAR at the CTC site show evidence of a local drainage flow with winds from the northwest that reach the Downtown area on this day (not shown). Mechanical turbulence and mixing of the near-surface layers, generated by the drainage flow, may explain the downward transport of power plant plumes, and resulting contributions to breathing level pollution.

#### b 30 to 31 January

This case is characterized by weak winds, strong temperature inversions^34^ and high surface pollution levels (see Figure 1). From 0300 to 0600 AKST on 30 January, large contributions (2–15 ppb) occur notably from the UAF A and B stacks (20 m), residing within the surface inversion on 30 January 0300 AKST. The strong signal (Figure 5b, panel (ii) between 20 and 50 m from 1200 AKST on 30 January that lasts for 24 h (low elevated layer up to 70 m on 30 January 1500 AKST, not shown) is due to emissions from the Aurora power plant simulated between surface and elevated inversion layers. The depth of the inversion layers increases over the following 12 h, and the simulated plume descends below the surface inversion layer (less than 50 m on 31 January 0300 AKST, not shown), leading to a surface contribution around midnight on 31 January (Figure 5b, panel iii). There are also influences from shorter stacks (Doyon, Zehnder and UAF). These results show that downward transport of power plant pollution is influenced by subsidence within shallow inversion layers. In this case, there is also some intermittent mixing, as suggested by δCO_2_. However, in contrast to 23 January, low δCO_2_ is not correlated to increased power plant contributions, and δCO_2_ values are smaller (0–30 ppm) than on 23 January.

Power plant tracer concentrations are also plotted as a function of observed δCO_2_ at breathing level during ALPACA-2022 (see Section S3 and Figure S3). Low (high) δCO_2_ correlates with strong (weak) power plant contributions under less (more) stable conditions. This supports the findings that enhanced vertical mixing leads to surface power plant enhancements, as seen on 23 January. On the other hand, low δCO_2_ sometimes correlates with lower contributions. In this case, increased mixing may also cause pollution to be lofted upward as well as transported downwind. As shown for the January 30–31 case, enhanced power plant concentrations also occur under very stable conditions. This is likely due to the subsidence of the power plant plumes that reside within layers close to the surface.

Overall, these results show observational evidence for downward transport of power plant pollution during ALPACA-2022 which is captured by the model simulations, including during stable meteorological conditions. They provide further confidence in the model simulations that were also validated in detail in Brett et al.^34^

### 3.3 Sensitivity to Stack Height and Stability

**Figure 6:**
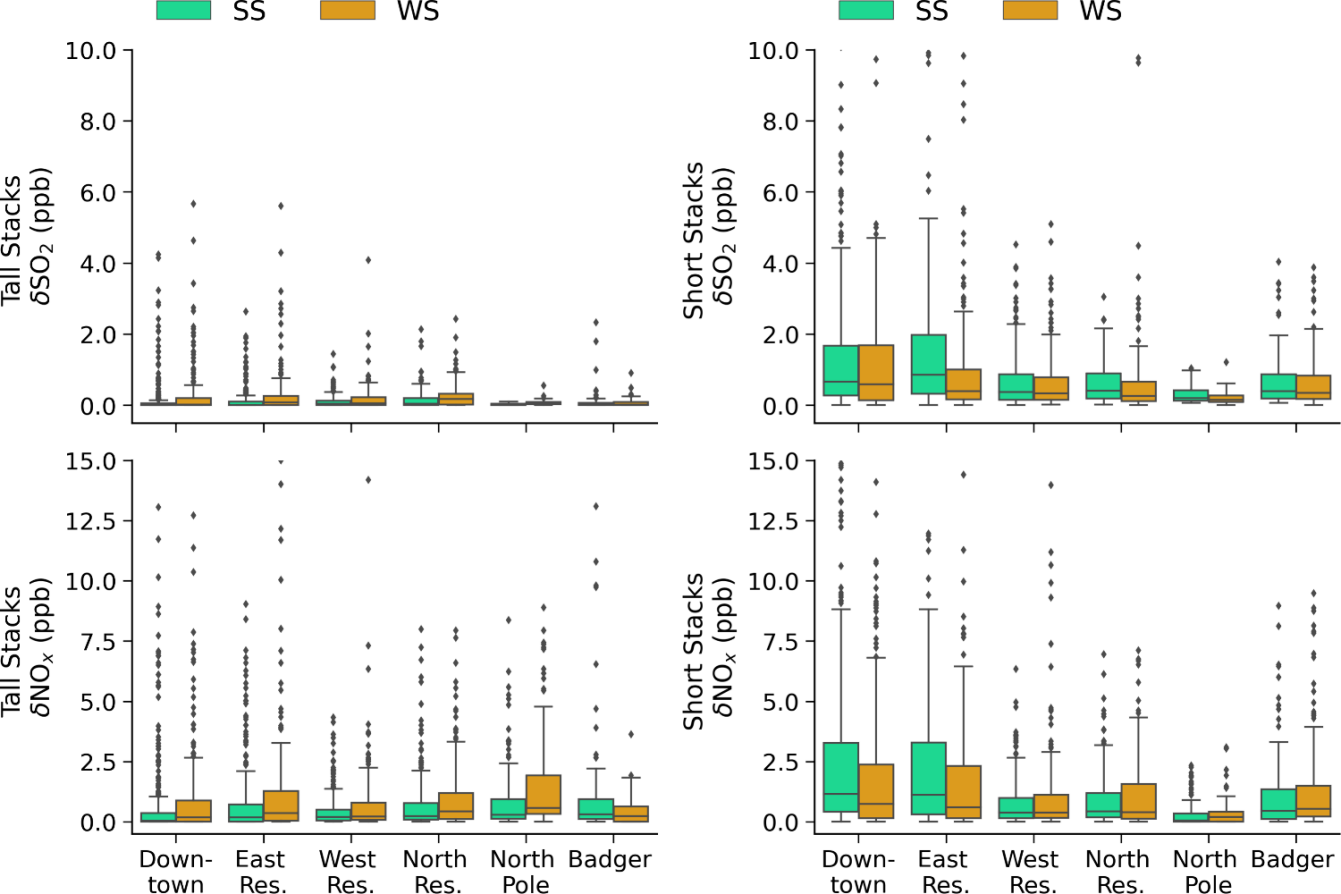
Hourly absolute power plant contributions (ppb) at breathing level for SS vs WS regimes averaged over each area for tall power plant stack heights (>30 m, left panels) and short power plant stack heights (<30 m, right panels) for (a) δSO_2_ and (b) δNO_*x*_. Box lower edge = 25th percentile, upper edge = 75th percentile, and middle line = 50th percentile (median). Lower whisker = lowest data point within 25th percentile minus the interquartile range (IQR) × 2, and upper whisker = 75th percentile plus IQR × 2. Scatter points are outliers.

**Figure 7:**
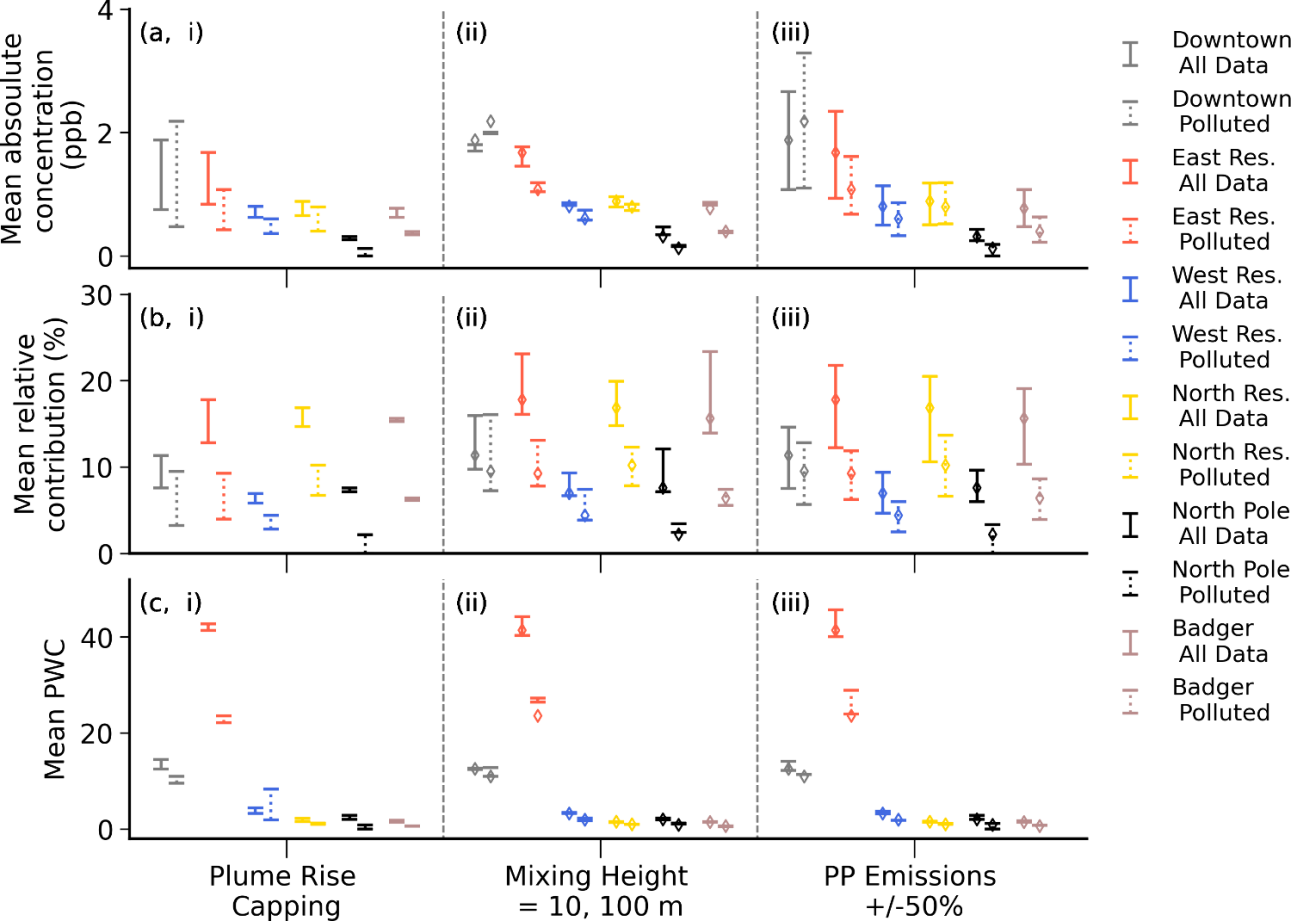
(a) δSO_2_ power plant concentrations (ppb) between 0 and 10 m for data when power plant concentrations >0.1 ppb. (b) δSO_2_ power plant contributions relative to the total δSO_2_ tracer (surface + power plant) in %. (c) SO_2_ population-weighted contributions (PWC) (see Section S1.3). Average values over the campaign (solid lines) and during ‘polluted periods’ (dashed lines) are shown. Results are shown for each area defined in Figure 2, and minimum and maximum values correspond to sensitivity simulations, see text for details regarding the upper and lower limits of the bars.

In this section, we investigate whether breathing level contributions are influenced by power plant stack heights under different stability regimes. Figure 4 demonstrates that power plant emissions contribute intermittently in each of the areas. For this reason, in the following analysis (Figures 6 and 7), results are evaluated when power plant enhancements are >0.1 ppb, which is 60 and 70% of the time for SO_2_ and NO_*x*_, respectively (Downtown), i.e. we evaluate periods when the model simulates a detectable surface influence from power plants in each area.

Figure 6 shows area average power plant contributions for tall stacks (above 30 m) and short stacks (below 30 m) under both SS and WS conditions. The 30 m limit is chosen based on the average surface based inversion height (34 m), since power plant emissions are often trapped above or below the surface inversion layers. In SS and WS conditions, the average surface inversion layer heights are similar, however the inversion strengths (temperature gradient,  per 100 m) vary (generally, WS: < 10 °C per 100 m; SS: 10–70 °C per 100 m, at 0–25 m altitude). While the 30 m threshold is used in all conditions, it is important to note that the inversion strength may influence whether power plant emissions penetrate the surface inversion layers as discussed earlier (see also discussion about the plume rise parametrization in Section S1.1).

The results show that power plant contributions (enhancements) at the breathing level are primarily due to power plants with shorter stacks, ranging between 0.0 and 3.2 ppb δSO_2_ and 0–5.1 ppb δNO_*x*_. These contribution ranges correspond to the lower and upper whiskers in Figure 6, and they exclude the outliers. δNO_*x*_ contributions are generally higher than δSO_2_ due to higher NO_*x*_ emissions from some of the main stacks, e.g. Aurora. They also show that enhanced contributions from shorter stacks are not dependent on stability regime. In contrast, contributions from taller stacks are larger during WS conditions (0.0–0.6 ppb δSO_2_ and 0.0–2.9 ppb δNO_*x*_), and smaller in SS conditions (0.0–0.3 ppb δSO_2_ and 0.0–1.9 ppb δNO_*x*_) when power plant emissions are generally simulated above shallow surface inversion layers, limiting downward transport. While previous studies investigating power plant contributions from tall stack heights (70 to 250 m), in regions other than the Arctic, also estimated small contributions to surface pollution,^16,25^ they did not consider the influence of variable stack heights. In one study using CALPUFF modeling of pollution from fossil fuel power plants in Illinois, it was assumed that down washing did not occur due to the height of the stacks (84–153 m).^40^ We demonstrate that this is not the case in Fairbanks during the winter.

This analysis shows that power plant emissions can be transported to the surface under both SS and WS meteorological conditions. The results also suggest that it is important to consider the stack height relative to the surface inversion layer heights. Notably, under SS conditions, shorter stacks contribute more to breathing level pollution. This is especially important in the Arctic wintertime ABL which is often highly stratified. The very limited previous studies examining the influence of power plant emissions in Fairbanks did not consider these aspects.^12,27^

### 3.4 Power Plant Contributions to Breathing Level Pollution

The absolute and relative power plant δSO_2_ tracer contributions to surface pollution, together with average PWC (population-weighted contributions) for SO_2_, over each area, are shown in Figure 7 (δNO_*x*_ results are shown in Figure S4). Results are shown as averages for the entire campaign, and during polluted conditions assigned using daily averaged observed PM_2.5_ > 15 μg m^–3^ from the NCore site (Downtown). This threshold is the World Health Organisation (WHO) 24-h air quality limit for PM_2.5_,^41^ and polluted days are shown in Figure 1. Results are also shown for the different sensitivity simulations described in Section S1.2 (and summarized in Table S2). This allows consideration of some of the uncertainties in the tracer simulations in stable wintertime stratified ABL conditions. In Figure 7, the simulations with and without power plant plume rise capping at inversion layers (CTRL and NOCAP) are the upper and lower limits of the error bars, respectively (panel i). The mixing height sensitivity is shown in panel ii (*h*_min_: 100 m = upper, 10 m = lower, CTRL = midpoint), and panel iii corresponds to the ± 50% power plant emission magnitude sensitivity (CTRL = midpoint).

The average absolute contributions are largest over the Downtown and East Residential areas (e.g., Downtown: 1.9 ppb (min-max: 0.8–2.7) ppb δSO_2_), where the first value corresponds to the CTRL simulation average and min-max corresponds to the sensitivity range. This translates to average relative contributions Downtown of 11% (min-max: 8–16%) SO_2_. These areas are in close proximity or downwind of the main power plants, notably, Doyon (see Figure 4). Previous studies in Kazakhstan with comparable stratified ABL conditions induced by topography,^24^ and Di Ciaula^42^ in Italy, showed greater contributions to near-surface pollution at locations near to power plants, although they did not evaluate surface power plant contributions relative to total surface emissions. The results for Fairbanks show surface influences that depend, not only on proximity to power plant facilities but also on prevailing winds, ABL stability, and downward mixing.

Absolute power plant contributions are higher in polluted conditions, Downtown (e.g., Downtown: 2.2 ppb (min-max: 0.5–3.3 ppb) δSO_2_), but not in other areas (Figure 7a). The relative contributions are lower in polluted conditions with respect to campaign averages (Figure 7b, e.g., Downtown: 9% (min-max: 3–16%) SO_2_) since power plant contributions become less important relative to total (power plant plus surface) emissions. This can be explained by enhanced near-surface emissions (Figure 1) when vertical dispersion is more limited in SS conditions, and emissions, notably from taller stacks, are often trapped above surface inversion layers that persist in such conditions, as discussed earlier. The mixing height sensitivity does not have a substantial impact on absolute contributions (Figure 7a, panel ii), but it does affect the relative contributions (Figure 7b, panel (ii). For instance, significant relative contributions (exceeding 20%) occur in East Residential, North Residential and Badger areas. This is because surface-emitted tracers are sensitive to the extent of vertical mixing below surface-based inversions (as explored in detail in Brett et al.^34^). In the run with *h*_min_ = 100 m, lower total tracer surface concentrations are simulated at the East and North Residential areas, compared to Downtown, due to increased vertical transport of surface pollution upward and power plant emissions downward. This results in higher relative power plant contributions to breathing level pollution (East Residential: 18% (min-max: 12–23%), North Residential 17% (min-max: 11–20%) SO_2_). The power plant capping sensitivity reveals increased contributions when capping of power plant emissions at surface or elevated inversion layers is applied, notably over the Downtown and East Residential areas (panel i).

Here, we compare our results with the only other previous study examining power plant contributions to surface pollution in Fairbanks.^12^ Analysis of CALPUFF dispersion modeling results for two 2-week (strongly and mildly polluted) episodes in 2008 estimated 4.4 and 9.7 μg m^–3^ SO_2_ on average from all point sources at the surface in downtown Fairbanks, corresponding to 10% and 21% relative contributions. As noted earlier, these simulations were evaluated against very limited observations. In contrast to the results presented here, ADEC^12^ showed that contributions at the surface were lower in the strongly polluted episode during stable conditions. While the average contribution from our study of 6.8 μg m^–3^ (9%) in polluted conditions (min-max: over Downtown from all power plants, falls within the CALPUFF estimates, our results demonstrate a large range 0.8–26.0 μg m^–3^ (2–22%)) δSO_2_. In addition, the absolute concentrations simulated in our study are higher under more stable conditions (Figure 6). Differences can be due to differences in modeling methodology, as well as the meteorology and emissions between the two years. It can be noted that the representation of boundary layer meteorology in the EPA-WRF simulations used to drive FLEXPART-WRF is improved for the ALPACA-2022 campaign, in part due to assimilation of observations.^43^ Also, while the CALPUFF model included plume rise for the power plant emission injection, it lacked consideration of the stably stratified conditions when emissions can be capped by inversion layers.^34^ The CALPUFF modeling study also did not include any deposition or chemical processing, and power plant emissions have changed since 2008. For example, SO_2_ emissions from Zehnder are very high compared to other stacks (1000 ppm of sulfur, Table S1)^35^ but, this stack was restricted to limited operation in winter 2022, and only ran intermittently, mainly during less polluted conditions (Figure 1). The largest average simulated δSO_2_ over Downtown is from the Doyon power plant, corresponding to a concentration of 2.4 μg m^–3^ under polluted conditions. This result is similar to estimated SO_2_ contributions from the Tuxpan power plant in Mexico (3.09 μg m^–3^).^15^ However, it is difficult to compare to such estimates that are annual averages in non-Arctic locations with meteorological conditions that are very different from those in Fairbanks in winter.

In Downtown, mean absolute contributions of δNO_*x*_ (4.4 ppb (min-max: 1.3–6.4 ppb)) generally exceed that of δSO_2_. However, relative contributions of NO_*x*_ (7% (min-max: 4–10%)) are lower, due to larger NO_*x*_ surface emissions from the mobile on-road and residential heating sectors (see Figure 2, and Brett et al.^34^). In contrast, the absolute and relative contributions of power plant NO_*x*_ are higher at the North Pole (Figure S4, e.g. CTRL = 0.3 ppb, 8% SO_2_ vs 1.6 ppb, 20% NO_*x*_), due to the dominant influence from the nearby North Pole A stack, which runs on naphtha fuel (high NO_*x*_ emissions^34^). These results are consistent with surface NO_*x*_ contributions associated with power plants based on an analysis of NO_2_ isotopic signatures (<18%), by Albertin et al.,^44^ for the Downtown area during ALPACA-2022. They illustrate the importance of taking different fuel types into account when simulating power plant effects on pollutant levels.

Relative contributions range from 6 to 23% and 5 to 28% (SO_2_ and NO_*x*_, respectively) in the East and North Residential, Badger and North Pole areas, with higher contributions depending on the species (see Figure 7 and Figure S4). However, for West Residential, relative contributions are lower (5–10% for both SO_2_ and NO_*x*_). This is because of its location in the main southwestern outflow where non power plant source contributions, including airport emissions, are higher than other suburban or residential areas. Figure 7 (panel c) also shows that PWCs are higher in Downtown and East Residential areas for each sensitivity because, as noted earlier, population densities are significantly higher compared to those in other areas (Table S3). Mobile sampling during ALPACA-2022 by Robinson et al.^45^ also identified large spatial gradients in the context of human exposure to PM_2.5_ levels. Levy et al.^40^ also quantified population-weighted concentrations due to 9 power plants in Illinois using CALPUFF dispersion modeling. The study differed from this one by investigating the influence of emissions on particulate constituents from power plants with taller stack heights (84–153 m, 8 of which were >100 m). Results were only analyzed on an annual basis showing relatively small contributions to the areas analyzed (population-weighted concentrations, annual averages = 0.04, 0.13 μg m^–3^ primary PM_2.5_ and secondary sulfate, respectively). Nevertheless, the findings revealed that a large number of people were exposed to the small concentration increments, with potentially significant human health implications.^40^

The analysis presented here provides the first detailed quantification of the influence of power plants on breathing level pollution in Fairbanks during wintertime, taking into account some of the uncertainties in the model simulations. The results show that areas in close proximity or downwind of power plants are the most affected at breathing level, as discussed earlier. They also reveal a dependence on the ABL stability and downward mixing. Absolute contributions are increased during polluted conditions, while relative contributions are reduced in polluted conditions due to limited dispersion of enhanced near-surface emissions. Relative contributions are generally lower for NO_*x*_ than SO_2_ owing to the increased magnitude of surface NO_*x*_ in central Fairbanks.

While surface pollution levels of the trace gases simulated here do not exceed daily averaged NAAQS during the ALPACA campaign, it can be noted that lower thresholds already exist. For example, WHO guidelines are currently 40 μg m^–3^ SO_2_ and 25 μg m^–3^ NO_2_ (daily averages).^41^ Such limits were generally exceeded on polluted days during ALPACA-2022 (not shown). The absolute power plant contributions simulated in this study are non-negligible on average over the campaign, with significant hourly variability, as discussed in Section 3.1. For example, more than 10 ppb of both SO_2_ and NO_*x*_ (approximately 30 and 22 μg m^–3^, respectively at −20 °C) is simulated on some days in the Downtown and East Residential areas (see Figure 4 and Figure S1). These trace gases are also precursors to the formation of secondary aerosols that contribute to PM_2.5_, that regularly exceeds the daily averaged NAAQS (35 μg m^–3^) during wintertime pollution episodes in the FNSB.

### 3.5 Estimates of Power Plant Contributions to Breathing Level Primary PM_2.5_

Here, simulated δSO_2_ results are used together with the ratio of primary PM_2.5_ to SO_2_ power plant emissions to estimate enhancements in primary PM_2.5_ at breathing level in Fairbanks. This can be considered as a lower limit of PM_2.5_ originating from power plants, since we do not take secondary aerosol production into account. This would require a more detailed aerosol modeling approach. See SI Section S5 for a detailed description of the methodology. Emission ratios of PM_2.5_ and SO_2_ are calculated from the ADEC power plant emissions for ALPACA-2022 for each stack,^34^ and multiplied by the modeled δSO_2_ surface enhancements to estimate power plant primary δPM_2.5_ due to power plants at breathing level. Primary PM_2.5_ emissions consist mainly of elemental carbon and other elemental particles (including selenium, aluminum and silica) and organic carbon (<25%), with a small contribution from primary sulfate (<20%), depending on the stack, and negligible primary nitrate and ammonium aerosol emissions (see Figure S5). Aurora and Doyon (run on coal) are the main emitters of primary PM_2.5_. The estimated daily mean absolute primary PM_2.5_ contribution associated with power plants in the Downtown area ranges from 0.1–1.1 μg m^–3^ (see Table S5 in SI) during polluted conditions, and 0.5 μg m^–3^ on average. These results are similar to López et al.^15^ who estimated 0.12 μg m^–3^ (0.0–1.43 μg m^–3^) for primary PM_2.5_ in Tuxpan, Mexico during 2001. Maximum hourly contributions are higher, especially in Downtown and East Residential, where they reach up to 6.3 μg m^–3^ of primary PM_2.5_ (see Figure S7). Other studies, in non-Arctic locations, found variable contributions to total PM_2.5_ (primary plus secondary) ranging from 0.0 to 4.3 μg m^–3^ depending on meteorology, emission controls and stack characteristics,^18,21,22^ however, primary PM_2.5_ contributions were not diagnosed separately. Relative primary PM_2.5_ contributions to total surface plus power plant pollution estimated here range from 0.6 to 6.2% (3.1% on average). ADEC^12^ estimated primary PM_2.5_ contributions from power plants to be <5% based on comparison with limited surface observations of total PM_2.5_, using CALPUFF modeling of the two pollution events (see earlier). They also did not evaluate the secondary PM_2.5_ production.

These estimates, while only for primary PM_2.5_, suggest that power plants in the FNSB are contributing to the aerosol burden at breathing level. This finding is supported by the LiDAR backscatter and Helikite profile observations collected during ALPACA-2022,^34^ although aerosol chemical composition of the plumes aloft is not available.^33^ PWC estimates for primary PM_2.5_ (not shown) indicate that East Residential is the main area affected. It is interesting to note that the newer, more efficient, UAF C stack has negligible primary PM_2.5_ emissions compared to the other stacks in Fairbanks (see Figure S6). However, while this stack uses limestone injection (wet flue gas desulfurization) to reduce SO_2_ emissions, it has been shown in China that this method significantly increases water vapor production leading to enhanced secondary aerosol production.^46^ Thus, the substantial SO_2_ and NO_*x*_ emitted by the Fairbanks diesel power plants (UAF A, B, Zehnder and North Pole A), relative to primary PM_2.5_, may also lead to significant secondary aerosol formation of sulfate and nitrate downwind of these stacks as shown by Levy et al.^40^ over the Chicago area (Illinois, midlatitudes).

## 4 Conclusions and Perspectives

High-resolution model simulations of trace gas enhancements above background from multiple power plant stack emissions and surface sector emissions are used to estimate absolute and relative power plant pollutant contributions to pollutant concentrations at breathing level (0–10 m) during ALPACA-2022. We use model simulations run with temporally varying emissions, power plant plume injection taking into account ABL stability, and validated and improved compared to campaign observations (detailed in Brett et al.^34^).

The analysis provides new insights into the factors influencing the transport of power plant emissions to the surface that were not considered in the limited previous Fairbanks studies.^12,27^ We show that contributions from power plants to breathing level pollutants display significant temporal variability across different stacks, ranging from 0.3 to 2.7 ppb (5–23% relative contribution) and 0.6 to 6.4 ppb (4–28% relative contribution), on average during ALPACA-2022, for SO_2_ and NO_*x*_, respectively. Higher absolute contributions for SO_2_ and NO_*x*_ occur in polluted conditions (0–3.3 and 0.2–9.1 ppb), but relative contributions are lower, 0–16% and 1–14%, due to enhanced surface pollution. Downtown and East Residential areas are most affected by power plant pollution at the surface (factor of 2 higher). These areas also have higher average population-weighted contributions (PWCs) due to higher population densities with respect to the FNSB. In contrast, relative contributions are generally higher (6–23% SO_2_ and 5 to 28% NO_*x*_) in other areas, where surface emissions are lower, indicating enhanced power plant influence on background pollution, relative to other sources, in suburban areas.

A key finding is that power plant emissions can be transported to the surface even in very stable conditions, such as the very cold polluted episode at the end of January 2022, in contrast to the findings of ADEC^12^ who estimated lower surface contributions in such conditions. Our results are based on analysis of the model simulations and observational evidence from ALPACA-2022. During SS conditions, power plant emissions from short stacks contribute more than those from tall stacks due to subsidence of power plant emission tracers trapped below the average surface inversion height (30 m in this study). Emissions from taller stacks may also be transported to the surface under SS conditions. For example, a local valley flow from surrounding hills may contribute to instabilities and down mixing in downtown Fairbanks, as shown for 23 January. Nevertheless, tall stacks contribute mainly in WS conditions due to more efficient vertical mixing induced by turbulence, whereas downward transport is generally inhibited during SS conditions.

These results provide new estimates of power plant contributions to surface pollution and new insights into processes that need to be considered. They emphasize the importance of taking into account temporal variations in power plant emissions, the complexities of wintertime ABL stability, and topographic effects affecting local flows, in addition to large-scale prevailing winds. The estimated contributions show significant sensitivity to the presence of surface temperature inversions and power plant stack height, highlighting the interplay between ABL stability and emissions, an important new finding. While previous studies examined horizontal and vertical dispersion of power plant plumes, they focused almost entirely on non-Arctic locations and seasons other than winter and did not consider these aspects. Moreover, planning for new power generation facilities in the Arctic needs to take into account proximity to local populations, as well as particularities related to Arctic boundary layer meteorology and topographical effects.

Simulated SO_2_ enhancements coupled with stack emission ratio data are also used to estimate primary PM_2.5_ power plant contributions at breathing level, which range between 0.1–1.1 (average = 0.5) μg m^–3^ (see Table S5 in SI) per day over the Downtown area during polluted conditions. These preliminary estimates are a lower limit since we do not take into account secondary aerosol production in power plant plumes. Nevertheless, primary PM_2.5_ alone makes important contributions to PM_2.5_ with significant hourly variability, reaching 6.3 μg m^–3^. It will be instructive to compare these results to more comprehensive chemical-aerosol modeling including primary (emitted) and secondary aerosols, although regional models may not capture pollution plumes as well as FLEXPART-WRF due to excessive diffusion related to 3D model resolution issues. The detection of trace metals, specific to power plants, could also contribute to improved estimates of power plant influence at the surface.

There is a need for future work investigating the influence of power plant and other emissions, such as space heating, released above the surface, over the wider Arctic during winter when stable boundary layers are a ubiquitous feature. It will be important to consider the factors highlighted in this study in estimates of elevated emission sources on breathing level pollution in other Arctic locations during the winter. For example, the height of power plant stacks or home heating emissions relative to surface stability criteria (frequency, depth, and strength of SBIs) will need to be examined. Power generation emissions in these vertically stratified environments may also be making important contributions to background trace gases and aerosol, notably wintertime Arctic haze, and potentially to aerosol-cloud indirect effects. Current emission inventories do not include, in particular, temporal variations in power plant emissions, and our results highlight the need for improved emission inventories of elevated sources in the Arctic. This, together with accurate simulation of the Arctic boundary layer, is needed to better quantify pollution impacts on Arctic air quality and climate.

## Supporting Information Available

Additional details on model simulations, sensitivity runs, and model simulation caveats. Calculations of PWCs and estimated primary PM_2.5_ concentrations explained in detail, and supporting figures(PDF)
