# Supplementary material for: Estimating Power Plant Contributions to Surface Pollution in a Wintertime Arctic Environment
Source: ACS EST Air. 2025 Apr 21;2(5):943–56. doi: 10.1021/acsestair.5c00030 (PMC12070414; doi:10.1021/acsestair.5c00030)
Supplement: Supplementary file 1 — ea5c00030_si_001.pdf [file ea5c00030_si_001.pdf]

Estimating power plant contributions to surface pollution in a wintertime Arctic environment - Supporting Information

Natalie Brett,[*,†,‡] Steve R. Arnold,[‡] Kathy S. Law,[†] Jean-Christophe Raut,[†] Tatsuo Onishi,[†] Brice Barret,[¶] Elsa Dieudonné,[§] Meeta Cesler-Maloney,[‖] William Simpson,[‖] Slimane Bekki,[†] Joel Savarino,[⊥] Sarah Albertin,[⊥,†,∇] Robert Gilliam,[#] Kathleen Fahey,[#] George Pouliot,[#] Deanna Huff,[@] and Barbara D'Anna[△]

†*Sorbonne Université, UVSQ, CNRS, LATMOS, 75252 Paris, France*

‡*Institute for Climate and Atmospheric Science, School of Earth & Environment, University of Leeds, LS2 9JT, UK*

¶*Laboratoire d'Aérologie (LAERO), Université Toulouse III – Paul Sabatier, CNRS, 31400 Toulouse, France*

§*Laboratoire de Physico-Chimie de l'Atmosphère (LPCA), Université du Littoral Côte d'Opale (ULCO), 59140 Dunkirk, France*

‖*Geophysical Institute and Department of Chemistry and Biochemistry, University of Alaska Fairbanks, Fairbanks, AK 99775, United States*

⊥*Univ. Grenoble Alpes, CNRS, IRD, INRAE, Grenoble INP, IGE, 38000 Grenoble, France*

#*Center for Environmental Measurement and Modeling, Office of Research and Development, US EPA, Research Triangle Park, NC 27709, United States*

@*Alaska Department of Environmental Conservation, P.O. Box 111800, Juneau, AK 99811-1800, United States*

△*Aix Marseille Univ, CNRS, LCE, 13331 Marseille, France*

∇*Currently at: Cooperative Institute for Research in Environmental Sciences, University of Colorado / Chemical Sciences Laboratory, National Oceanic and Atmospheric Administration, Boulder, CO 80305, USA*

E-mail: natalie.brett@latmos.ipsl.fr

Contents

S1: Methodology **3**

S1.1 FLEXPART-WRF simulations 3

S1.2: Sensitivity simulations 5

S1.3 Population weighted contributions 6

S2: Spatial contributions **7**

S3: Influence of vertical mixing and stability **10**

S4: Power plant contributions to breathing level pollution **12**

S5: Primary $PM_{2.5}$ estimates **13**

S1: Methodology

S1.1 FLEXPART-WRF simulations

FLEXPART-WRF tracer simulations were run at 1.33 km horizontal resolution up to 500 m altitude. The effects of local topography are included via the driving wind fields that come from the high resolution EPA-WRF simulation. Multiple particles corresponding to CO, SO_2, NO and NO_2 tracers were simulated. 80,000 particles were released hourly for near-surface emission sources (total) at 0-4, 4-8 and 8-12 m altitude at each grid cell, with masses weighted according to emissions from the Alaska Department of Conservation (ADEC) ALPACA-2022 inventory (also 1.33 km resolution). 5000 particles for each power plant stack (8 stacks from 18-64 m high) were released hourly as point sources at the location and plume rise height according to the plume rise parameterization in Brett et al. [1]. Emission masses of power plants were weighted according to the emissions during ALPACA-2022 provided by the power plant companies. For the Doyon stack 2020 data was used because 2022 data was not provided. Results for CO are not discussed in this study since CO power plant emissions are very low with respect to the near-surface emitted sources. Information on the power plant stack heights, fuel type and emission controls with regard to sulfur and nitrogen emissions are provided in Table S1.

The plume rise parameterization includes capping depending on the occurrence of surface or elevated temperature inversions layers, diagnosed from 12-hourly radiosonde observations during ALPACA-2022. The model results were generally optimal in the capping simulation, as shown by comparison against vertical profiles of trace gases measured at the UAF Farm, downwind of power plant influence aloft (80-300 m). At the surface, model results are sensitive to the minimum mixing height parameter ($\mathbf{h}_{\min}$) in FLEXPART-WRF which represents vertical mixing. This is important for simulating breathing level concentrations, notably for SO_2 which has space heating emissions extending above the surface level. Although the results are dependent on the surface stability and meteorology, biases in simulated trace

Table S1: Power plant characteristics. 'A', 'B' and 'C' denote separate burners and stacks at the same power plant facility. The power plant locations are shown in Figure 2 (main text). The emission control information for nitrogen (N) and sulfur (S) emissions is summarized from Brett et al.[1] and more details are provided in ADEC[2].

Power Plant	Stack Height (m)	Fuel Type	Emission controls
Aurora	48	Coal	**S:** 0.25 % sulfur by weight
Zehnder	18	Diesel	**S:** 1000 ppm sulfur, limited operations (<70 tons per year)
UAF A	20	Diesel	**S:** 15 ppm sulfur
UAF B	20	Diesel	**S:** 15 ppm sulfur
UAF C	64	Coal	**S:** 0.25 % sulfur by weight, limestone injection; **N:** low NO_x burners & staged combustion.
Doyon	26	Coal	**S:** 0.25 % sulfur by weight
North Pole A	34	Naphtha	**S:** 50 ppm sulfur; **N:** limited operations
North Pole B	19	Diesel	**S:** 15 ppm sulfur; **N:** limited operations

gases are generally optimal when $\mathbf{h}_{\min} = 20$ m.[1] More details about the tracer simulations, emissions, and the plume rise parameterization are provided in Brett et al.[1]. Results from the CTRL simulation (20 m $\mathbf{h}_{\min}$, and including plume rise capping) are used in Figures 3-7 in the main text. The exception is the Zehnder stack, where the capping simulation is used only in strongly stable (SS) conditions, and not in weakly stable (WS) conditions. This is because capping leads to large overestimates in surface concentrations with respect to observations in WS conditions (not shown). Zehnder has a low stack (18 m) and high emissions,[2] however the parameterization often caps Zehnder plumes when a weak SBI occurs, highlighting a deficiency in the parameterization (see discussion in Brett et al.[1]). Future studies should aim to include plume penetration of the SBI layer to improve the plume rise capping parameterization.

S1.2: Sensitivity simulations

Sensitivities of breathing level power plant contributions are explored in Section 3.3 (main text). This includes sensitivity to plume rise capping (switched on (CTRL) and off (NOCAP)), vertical mixing ($\mathbf{h}_{\mathrm{min}}$ values), and power plant emission magnitudes (± 50%), as summarized in Table S2.

Table S2: CTRL and sensitivity simulations used in this study.

Sensitivity Type	CTRL Description	Sensitivity Runs
Plume Rise Capping	**CTRL**: Briggs plume rise plus capping at inversion layers and NOCAP for Zehnder in WS conditions.	**NOCAP**: Briggs plume rise, capping at inversion switched off.
Vertical Mixing	**CTRL**: $\mathbf{h}_{\mathrm{min}} = 20$ m	$\mathbf{h_{min} = 10}$ **and 100 m** (both power plants and near-surface tracers).
Power Plant Emissions	**CTRL**: $\mathbf{h}_{\mathrm{min}} = 20$ m	± **50%** power plant emission magnitudes for SO_2 and NO_x tracers.

The results presented here could be further refined with improved simulation of the atmospheric boundary layer (ABL) in Arctic wintertime conditions, and notably the presence of surface and elevated inversion layers in the WRF model, used to drive FLEXPART-WRF. The plume rise parameterization could also be improved by using hourly WRF vertical profiles implemented in online calculations, to increase temporal and spatial coverage.[1] Finally, validating power plant emission magnitudes, such as with drone measurements downwind of plumes and close to stacks, could reduce the need for the ±50 % power plant emission sensitivity. Further discussion regarding model uncertainties and potential future improvements is provided in Brett et al.[1].

S1.3 Population weighted contributions

The population-weighted contributions (PWCs) are calculated according to concentration ratios and weight factors in each area or in each grid cell (based on e.g. López et al.[3], Zhang et al.[4], Prasannavenkatesh et al.[5]). The weight factors (Equation 2) are calculated according to the ratio of population densities (Equation 1). The values are shown in Table S3 for FNSB and each of the analysis areas shown in Figure 2 in the main text. The overall PWCs are ratios of the mean concentration in the area or grid cell with respect to the FNSB mean, multiplied by the weight factor (Equation 3).

$$\rho_i = \frac{P_i}{A_i} \tag{1}$$

Where i = grid cell or sum of grid cells in the area, P = population total in the area or grid cell, A = area in km^2 and ρ = population density (units = people km^{-2})

$$WF_i = \frac{\rho_i}{\rho_{FNSB}} \tag{2}$$

Where WF is the weight factor, a unit-less ratio and ρ_{FNSB}, is it the population density in the FNSB non-attainment area (people km^{-2}). This method is based on Zhang et al.[4].

$$PWC_i = \frac{\overline{\sum C_i}}{\overline{\sum C_{FNSB}}} \times WF_i \tag{3}$$

Where $\overline{\sum C_i}$ is the mean concentration in the area of interest or grid cell and $\overline{\sum C_{FNSB}}$ is the mean concentration in the FNSB area.

Table S3: Total population counts, number of model cells, population density and weight factors for FNSB and each analysis area shown in Figure 2 (main text).

Area Name	Total Population	No. grid cells	Population Density (ρ) (people km^{-2})	Weight Factor (WF)
FNSB	78644	339	130	1.0
Downtown	2785	6	261	2.0
East Residential	9569	4	1346	10.3
West Residential	1586	9	99.2	0.76
North Residential	3190	15	120	0.92
North Pole	4548	9	284	2.18
Badger	1908	9	119	0.91

S2: Spatial contributions

Time series of δNO_x power plant concentrations between 0-10 m are shown in Figure S1 (as for δSO_2 in Fig. 4 in the main text). Polar plots of total power plant δSO_2 and δNO_x concentrations as a function of wind direction and wind speed are shown in Figure S2. The filled coloured circles correspond to data >0.1 ppb, in order to highlight when concentrations become more important. The colourless circles show data <0.1 ppb. Figure S2 highlights the main wind directions bringing power plant tracers to each of the analysis locations. The North and East Residential areas are influenced by winds from the south/south-west (from Aurora, Zehnder and UAF, stacks a-c in Figure 2), and by winds from the east/north-east

(from Doyon and North Pole, stacks d-e) with greater concentrations at lower wind speeds (0-4 m s^{-1}). In contrast, Downtown and West Residential areas are predominantly influenced by winds from the east/north-east (from stacks a-d) at wind speeds up to 8 m s^{-1}. In the Badger area winds from the north-east correspond to low contributions, while winds from the west/north-west up to 4 m s^{-1} (from stacks a-d) lead to higher contributions (up to 5 ppb δSO_2). North Pole is influenced by winds from the east/north-east for δSO_2 (stacks a-d) but contributions are small. Larger δNO_x contributions (up to 10 ppb) are dominated by winds from the south (0-2 m s^{-1}) and south-west of up to 10 m s^{-1} (from stack e).

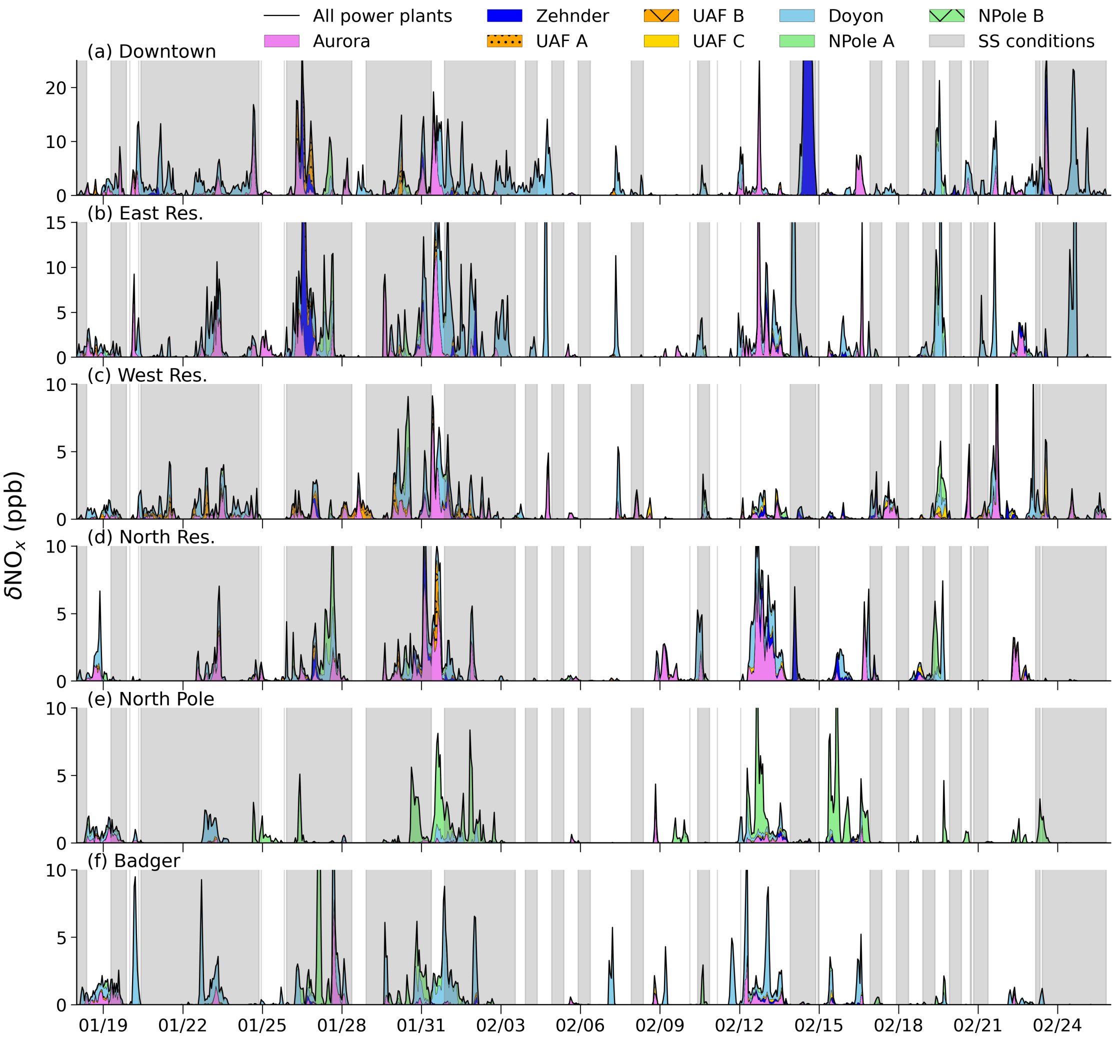

Figure S1: Total power plant δNO_x (ppb) concentrations (black lines) between 0-10 m as a function of time (AKST), colored by the different power plant contributions, indicated in the legend. Panels (a) to (f) correspond to the different analysis areas, indicated in Figure 2 (main text). The grey shaded periods correspond to SS conditions and the non-shaded periods correspond to WS conditions. See main text.

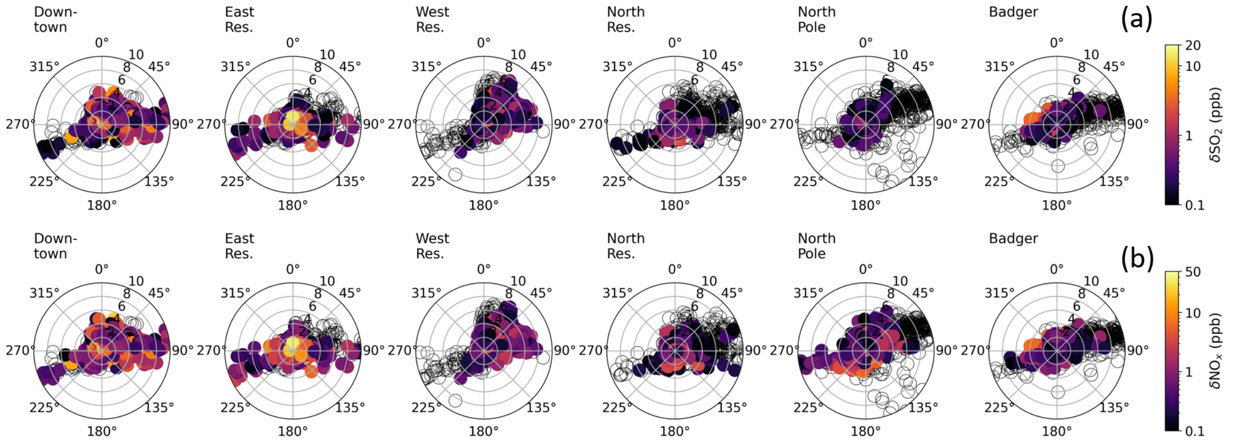

Figure S2: Hourly power plant δSO_2 (a) and δNO_x (b) concentrations (ppb) between 0-10 m altitude plotted as a function of wind direction and wind speed for the ALPACA campaign for each of the areas discussed in the main text. The colourless circles correspond to values below 0.1 ppb. Circular contours correspond to wind speeds (from 0-10 m s^{-1}) and the wind directions are indicated with 0 degrees (N) at the top. Wind variables at approximately 40 m, averaged over each area, are taken from EPA-WRF simulations used to drive the FLEXPART-WRF tracer simulations[1].

S3: Influence of vertical mixing and stability

Correlations between total power plant tracer concentrations (Downtown) and observed hourly 3-23 m CO_2 (δCO_2) during ALPACA-2022 are shown in Figure S3 (panels a-c). Panel (a) shows the kernel density correlation plot. In panels (b) and (c), the correlation scatter points are coloured by 23-3 m temperature differences at the CTC location and by the relative power plant contributions (%), respectively. Small δCO_2 values are representative of less stable conditions, and may indicate downward transport of pollution plumes to breathing level, since CO_2 is mainly from surface sources. Simulated power plant contributions sometimes increase at low δCO_2 (panels a and c), indicating stronger vertical mixing. However, on other occurrences, power plant enhancements are 0, or close to 0, possibly due to strong horizontal winds or turbulence, causing the power plant tracers to be lofted upwards or downwind,[1] rather than towards the surface. Enhanced power plant contributions sometimes occur during strongly stable conditions, shown by larger observed 23-3 m tem-

perature values in panel (b) e.g. between 1-7 ppb. But this is generally coupled with higher δCO_2 values, suggesting subsidence of the power plant tracers that reside within layers close to the surface, as indicated in case study (b) of the main text.

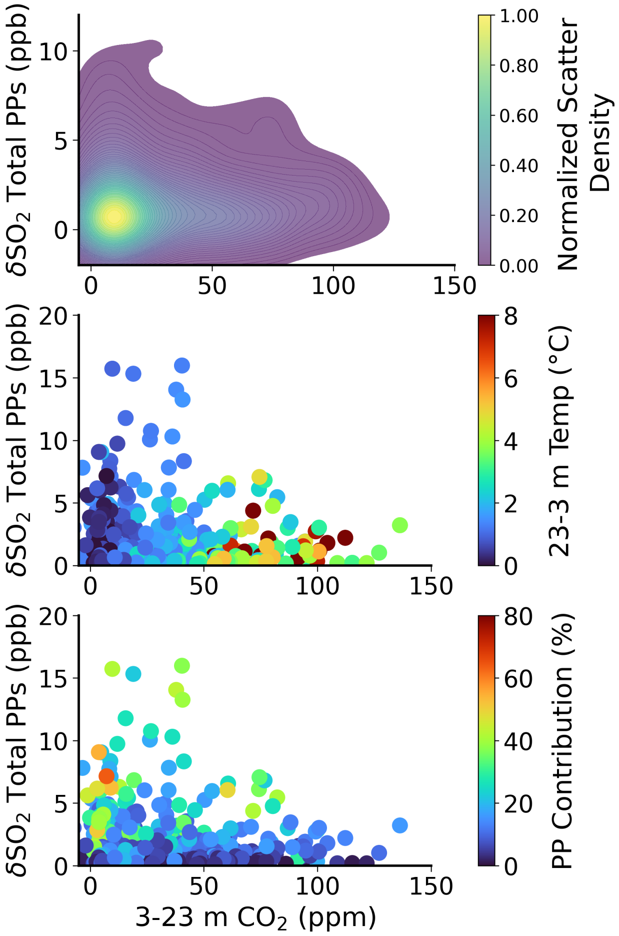

Figure S3: Hourly total power plant (PP) tracer δSO_2 concentrations (ppb), for the Downtown area, as a function of 3-23 m δCO_2 (ppm) (at CTC), presented as a) kernel density plot (normalized scatter density), b) scatter plot coloured by observed 23 - 3 m temperatures (°C) (CTC) and c) scatter plot coloured by relative power plant contributions (%).

S4: Power plant contributions to breathing level pollution

For each of the sensitivity runs described in Table S2, the absolute and relative power plant δNO_x tracer contributions, together with average PWCs (population-weighted contributions) for NO_x in each area are shown in Figure S4 (as in Figure 7 for δSO_2, main text).

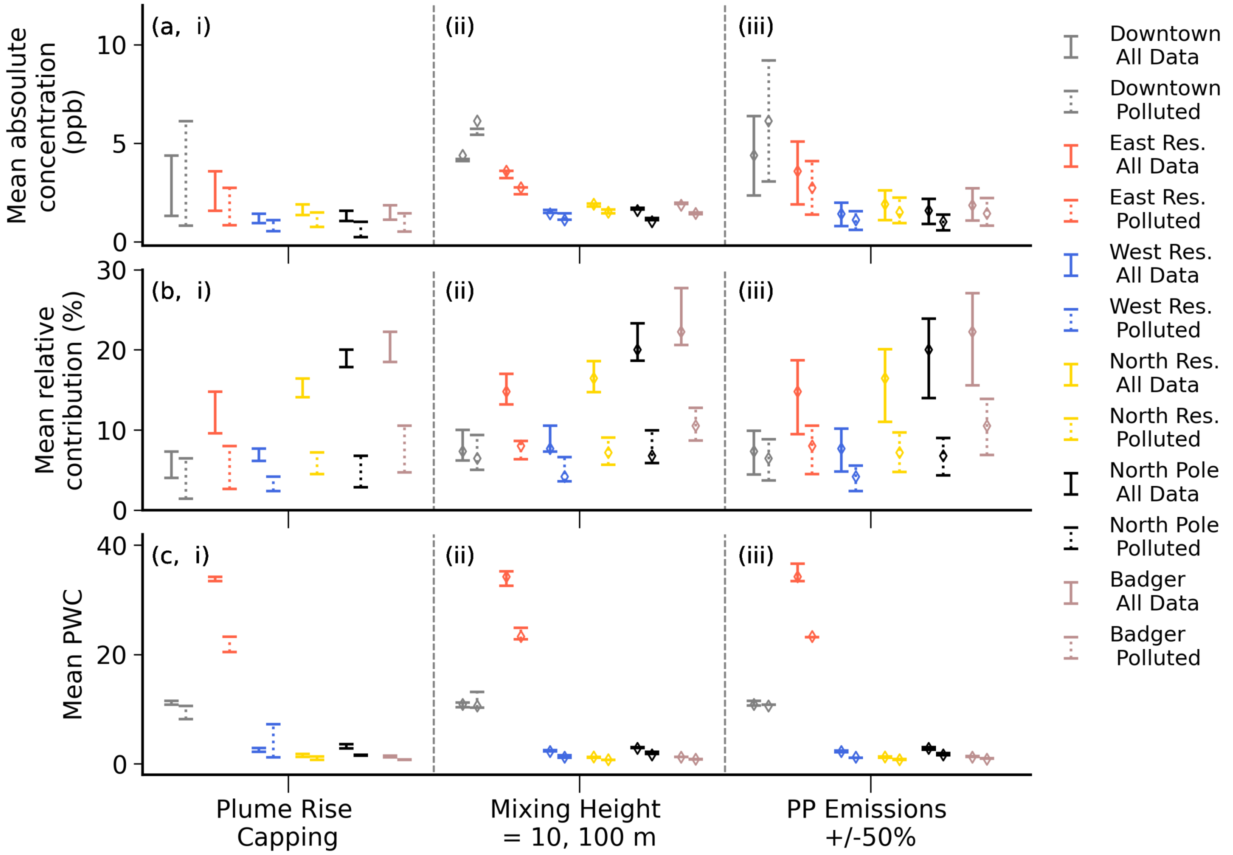

Figure S4: a) δNO_x power plant concentrations (ppb) between 0-10 m for data when power plant concentrations >0.1 ppb, b) δNO_x power plant contributions relative to the total δNO_x tracer (surface + power plant) in %, and c) NO_x PWCs (see Section S1.2). Average values over the campaign (solid lines), and during 'polluted periods' (dashed lines) are shown. Results are shown for each area defined in Figure 2 (main text), and minimum and maximum values correspond to sensitivity simulations, see Section 3.4 of main text for details.

S5: Primary $PM_{2.5}$ estimates

In this section, primary particulate matter (<2.5 μm) ($PM_{2.5}$) enhancements from power plants at breathing level are estimated using the ratio of power plant emissions of primary $PM_{2.5}$ and SO_2, multiplied by the FLEXPART-WRF results for δSO_2 at breathing level. First, the primary $PM_{2.5}$ emissions are discussed followed by a description of the methodology, results and caveats.

Information regarding the primary aerosol emissions for the power plants are provided in Fig. S5. According to the emission inventory, the Aurora and Doyon coal power plants produce the most total primary PM emissions, followed by North Pole A (panels a and b). The diesel power plants: UAF A, B and Zehnder emit relatively low primary PM, while negligible contributions are due to the UAF C stack, which is the newer more efficient power plant stack (more stringent regulations[1]). The primary aerosol emission contributions are shown in panel (c). Each aerosol species is considered as fine aerosol ($PM_{2.5}$), excluding anthropogenic coarse mode aerosol (PM_{10}). Other unspeciated aerosol is one of the key contributors and includes other elemental particles including selenium, aluminium and silica,[6,7] and primary sulfate only contributes to a small fraction of total primary $PM_{2.5}$.

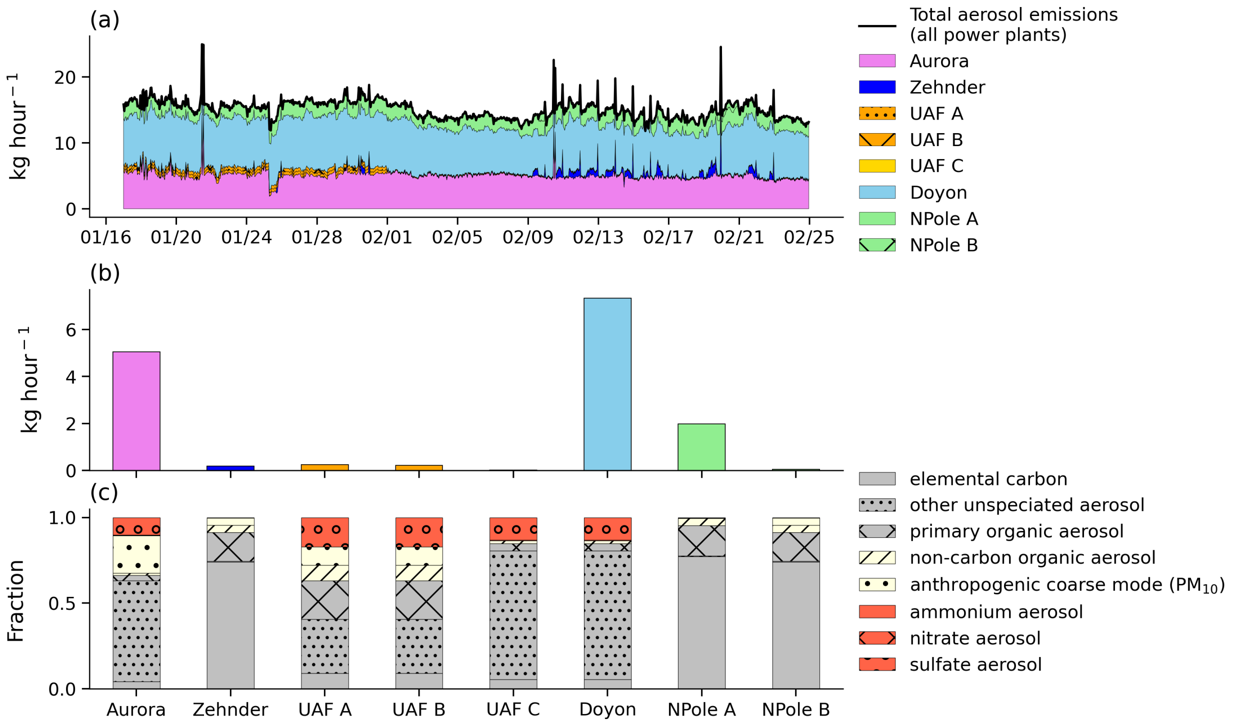

Figure S5: (a) Time series of total aerosol emissions (kg hour^{-1}) (black line = total power plants), according to each power plant stack (colors), (b) total average emissions for each power plant stack (kg hour^{-1}), (c) fraction of aerosol species for each power plant stack.

Table S4: Average primary PM_{10} emissions and $PM_{2.5}$ emissions (excluding anthropogenic coarse mode) (kg hour^{-1}), SO_2 emissions (kg hour^{-1}), and PM_{10}/SO_2 and $PM_{2.5}/SO_2$ emission ratios for the power plant stacks.

	Aurora	Zehnder	UAF A	UAF B	UAF C	Doyon	NPole A	NPole B	Total
PM_{10}	5.04	0.185	0.238	0.211	0.016	7.283	1.974	0.049	15.0
$PM_{2.5}$	3.94	0.177	0.212	0.189	0.016	7.283	1.974	0.047	13.8
SO_2	26.1	5.00	7.05	6.27	8.85	57.0	0.397	0.418	111
PM_{10}/SO_2	0.193	0.037	0.034	0.034	0.002	0.128	4.97	0.117	0.135
$PM_{2.5}/SO_2$	0.151	0.035	0.03	0.03	0.002	0.128	4.97	0.112	0.125

Power plant enhancements in $PM_{2.5}$ ($\delta PM_{2.5}$) between 0-10 m are estimated using the ratio of primary $PM_{2.5}/SO_2$ emissions for the individual power plant stacks. Primary $PM_{2.5}$

emissions are defined as the total primary aerosol minus the coarse mode anthropogenic fraction (see panel c in Fig. S5) since the goal is to estimate contributions for the criteria pollutant $PM_{2.5}$. The campaign-average primary $PM_{2.5}$ and SO_2 emissions for the individual power plant stacks are shown in Table S4. They are used to estimate the $PM_{2.5}/SO_2$ emission ratios. Since the ratio is constant throughout the campaign, the average values are used. Table S4 also shows total primary PM_{10} contributions including the coarse mode fraction. Most of the coarse-mode particles are emitted by the Aurora stack, but the contribution is relatively small. Moreover, the total power plant PM_{10}/SO_2 and $PM_{2.5}/SO_2$ ratios are comparable (0.135 and 0.125, respectively). This is likely because many of the larger particles are filtered by aerosol filters (bag houses) resulting in mainly fine particles ($PM_{2.5}$), according to the emission inventory.

The daily mean $\delta PM_{2.5}$ contributions are estimated using daily simulated δSO_2, multiplied by the $PM_{2.5}/SO_2$ emission ratio for each power plant stack (Table S4). This approach assumes that primary $PM_{2.5}$ and SO_2 directly emitted by power plants have residence times long enough to be treated as approximate tracers, or at least that they are transported and removed at a similar rate. The dominant removal process is assumed to be dry deposition for both species. Dry and wet deposition, as well as SO_2 oxidation, are taken into account in the SO_2 simulations, albeit in a simplified manner, but they contribute only small losses overall. Based on the ALPACA-2022 emissions for primary $PM_{2.5}$, the main contributions are from primary elemental or organic carbon (Fig. S5) that are also expected to be subject to dry deposition. Hence, as a first approach, modeled SO_2 enhancements and the emission ratios can be used to estimate primary $PM_{2.5}$ from power plants at the surface.

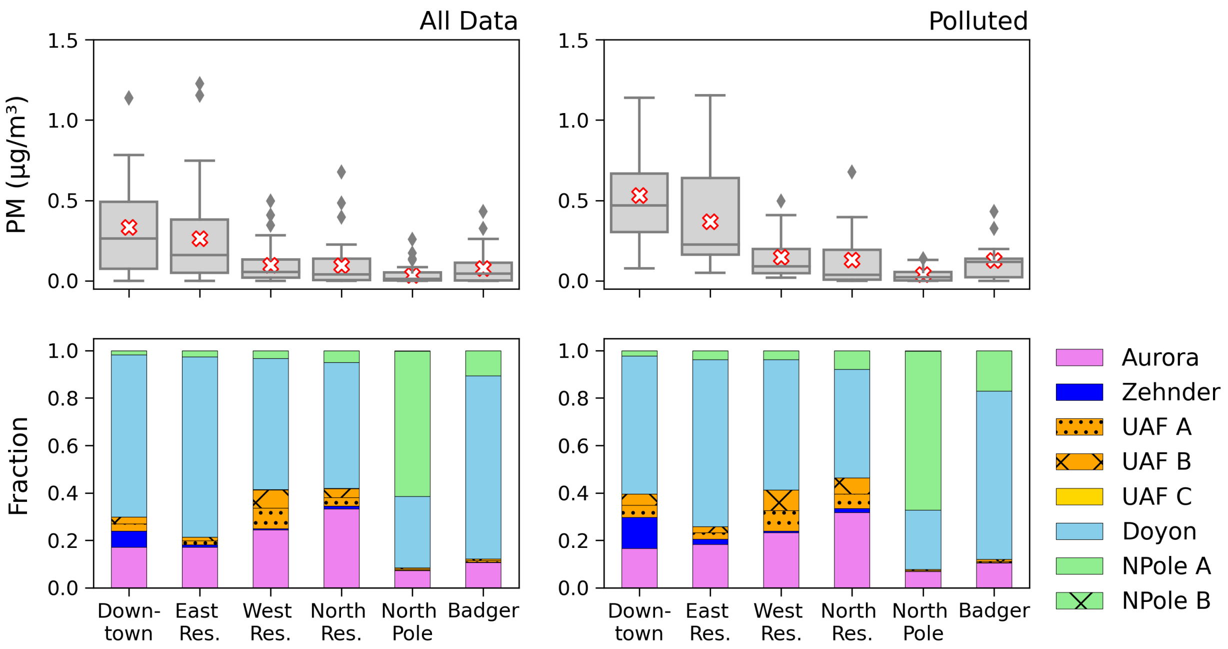

Figure S6: Daily averaged estimated values of $\delta PM_{2.5}$ ($\mu g\, m^{-3}$) associated with power plants in each area (top panels), for the full campaign (left) and polluted periods (right). The fractional contributions for each power plant are shown in the lower panels.

The results are presented in Fig. S6 for each of the areas defined in the main text (top panels). The red crosses correspond to the mean values, which are also given in Table S5 for the Downtown area. The lower panels show the power plant stack contributions as a fractions between 0 and 1. The results are also discussed in the main text, Section 3.5. The Doyon stack contribution dominates in the Fairbanks areas. This is expected due to the higher $PM_{2.5}/SO_2$ ratio. Furthermore, the Doyon stack is shorter than Aurora, and thus makes larger surface contributions. The Aurora power plant is the next largest contributor in all areas (excluding North Pole). Hourly contributions for each area are shown in Fig. S7. Significant intermittent contributions are estimated reaching up to 6.3 $\mu g\, m^{-3}$ (East Residential) and 4.2 $\mu g\, m^{-3}$ (Downtown).

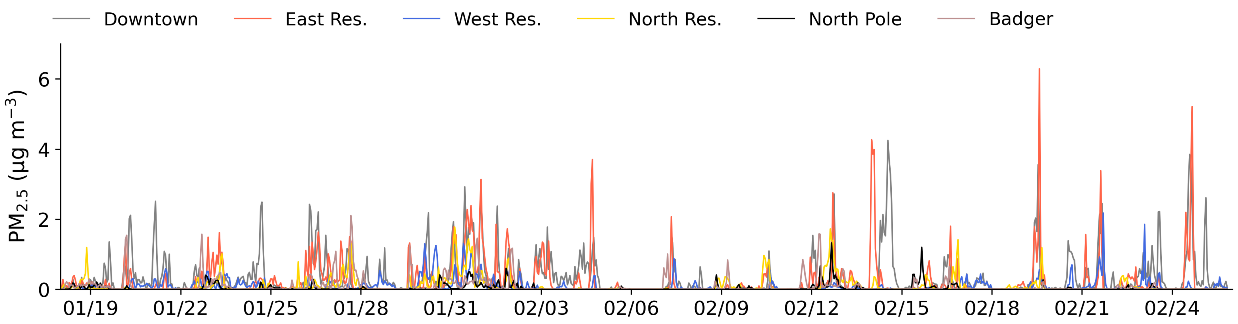

Figure S7: Hourly estimated values of primary $\delta PM_{2.5}$ ($\mu g\, m^{-3}$) associated with power plants for each area.

Relative contributions of estimated primary $\delta PM_{2.5}$ to observed $PM_{2.5}$ are calculated for the Downtown area and discussed in the main text. Total observed $PM_{2.5}$ includes primary and secondary $PM_{2.5}$ from surface and elevated sources. The aim here is to understand if power plant sources are making a notable contribution to breathing level $PM_{2.5}$, the main EPA criteria pollutant. However, this represents a lower limit since secondary aerosol production is not accounted for (see discussion in the main text). The simulated mean, min and max daily values are shown in Table S5 for the full campaign and polluted conditions (Downtown). The mean primary $\delta PM_{2.5}$ increases slightly in polluted conditions, as also depicted in Fig. S6 (panel b) in the Downtown and East Residential areas. The maximum relative contribution decreases in polluted conditions, despite increased absolute concentrations, consistent with SO_2 and NO_x enhancements due to power plants. The PM_{10} contributions are also shown in Table S5, to demonstrate that most of the primary PM from power plants is in the form of $PM_{2.5}$.

Table S5: Mean, maximum and minimum estimated primary power plant PM_{10} and $PM_{2.5}$ ($\mu g\,m^{-3}$) concentration enhancements and relative contributions (to observations) between 0-10 m in the Downtown area, for the full campaign and polluted conditions (24-hour averaged).

Species (averaging type)	Concentration ($\mu g\,m^{-3}$)			Relative Contribution		
	Mean	Max	Min	Mean	Max	Min
PM_{10} (campaign)	0.4	1.3	0.0	3.3 %	8.9 %	0.1 %
$PM_{2.5}$ (campaign)	0.3	1.1	0.0	3.1 %	8.9 %	0.1 %
PM_{10} (polluted)	0.6	1.3	0.1	3.3 %	6.5 %	0.6 %
$PM_{2.5}$ (polluted)	0.5	1.1	0.1	3.1 %	6.2 %	0.6 %

References

(1) Brett, N.; Law, K. S.; Arnold, S. R.; Fochesatto, J. G.; Raut, J.-C.; Onishi, T.; Gilliam, R.; Fahey, K.; Huff, D.; Pouliot, G.; others Investigating processes influencing simulation of local Arctic wintertime anthropogenic pollution in Fairbanks, Alaska, during ALPACA-2022. *Atmospheric Chemistry and Physics* **2025**, *25*, 1063–1104.

(2) ADEC *Amendments to: State Air Quality Control Plan, Vol. II: III.D.7.07 Control Strategies*; Alaska Department of Environmental Conservation (ADEC), 2020; accessed on 2 December 2024 from: `https://dec.alaska.gov/`.

(3) López, M. T.; Zuk, M.; Garibay, V.; Tzintzun, G.; Iniestra, R.; Fernández, A. Health impacts from power plant emissions in Mexico. *Atmospheric environment* **2005**, *39*, 1199–1209.

(4) Zhang, Y.; Tao, S.; Shen, H.; Ma, J. Inhalation exposure to ambient polycyclic aromatic hydrocarbons and lung cancer risk of Chinese population. *Proceedings of the National Academy of Sciences* **2009**, *106*, 21063–21067.

(5) Prasannavenkatesh, R.; Andimuthu, R.; Kandasamy, P.; Rajadurai, G.; Kumar, D. S.; Radhapriya, P.; Ponnusamy, M. Assessment of population exposure to coarse and fine particulate matter in the urban areas of Chennai, India. *The Scientific World Journal* **2015**, *2015*.

(6) Reff, A.; Bhave, P. V.; Simon, H.; Pace, T. G.; Pouliot, G. A.; Mobley, J. D.; Houyoux, M. Emissions inventory of PM2. 5 trace elements across the United States. *Environmental science & technology* **2009**, *43*, 5790–5796.

(7) ADEC *Amendments to: State Air Quality Control Plan, Vol. II: III.D.7.8, Modeling, Public Review Draft*; Alaska Department of Environmental Conservation (ADEC), 2024; accessed on 12 December 2024 from: https://dec.alaska.gov/.
